# Au@AuPd Core-Alloyed Shell Nanoparticles for Enhanced Electrocatalytic Activity and Selectivity under Visible Light Excitation

**DOI:** 10.1021/acsnano.4c07076

**Published:** 2024-08-20

**Authors:** Kaline N. da Silva, Shwetha Shetty, Sam Sullivan−Allsop, Rongsheng Cai, Shiqi Wang, Jhon Quiroz, Mykhailo Chundak, Hugo L. S. dos Santos, IbrahiM Abdelsalam, Freddy E. Oropeza, Víctor A. de la Peña O’Shea, Niko Heikkinen, Elton Sitta, Tiago V. Alves, Mikko Ritala, Wenyi Huo, Thomas J. A. Slater, Sarah J. Haigh, Pedro H. C. Camargo

**Affiliations:** † Department of Chemistry, University of Helsinki, A.I. Virtasen aukio 1, PO Box 55, FIN-0014 Helsinki, Finland; ‡ Department of Materials, University of Manchester, Manchester M13 9PL, United Kingdom; § Photoactivated Processes Unit, IMDEA Energy Institute, Avda. Ramón de la Sagra 3, 28935 Mostoles, Madrid, Spain; ∥ VTT Technical Research Centre of Finland, P O Box 1000, FIN-02044 Espoo, Finland; ⊥ Department of Chemistry, Federal University of Sao Carlos, Rod. Washington Luis, km 235, Sao Carlos 13565−905, Brazil; # Departamento de Físico-Química, Instituto de Química, Universidade Federal da Bahia, Rua Barão de Jeremoabo, 14740170-115 Salvador, BA, Brazil; ∇ College of Mechanical and Electrical Engineering, Nanjing Forestry University, Nanjing 210037, P. R. China; ○ NOMATEN Centre of Excellence, National Centre for Nuclear Research, Otwock 05-400, Poland; ◆ Cardiff Catalysis Institute, School of Chemistry, Cardiff University, Cardiff CF10 3AT, United Kingdom

**Keywords:** plasmonic electrocatalysis, nitrite reduction reaction (NO_2_RR), bimetallic nanoparticles, Au@AuPd core−shell, selectivity, visible light irradiation, ultralow loading

## Abstract

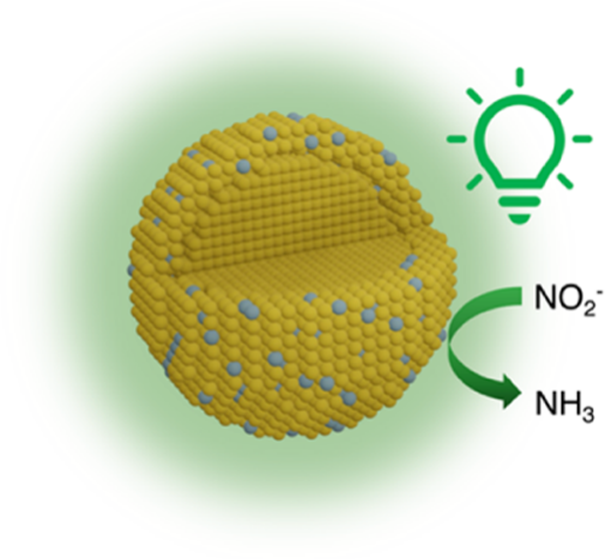

Plasmonic catalysis has been employed to enhance molecular transformations under visible light excitation, leveraging the localized surface plasmon resonance (LSPR) in plasmonic nanoparticles. While plasmonic catalysis has been employed for accelerating reaction rates, achieving control over the reaction selectivity has remained a challenge. In addition, the incorporation of catalytic components into traditional plasmonic-catalytic antenna-reactor nanoparticles often leads to a decrease in optical absorption. To address these issues, this study focuses on the synthesis of bimetallic core@shell Au@AuPd nanoparticles (NPs) with ultralow loadings of palladium (Pd) into gold (Au) NPs. The goal is to achieve NPs with an Au core and a dilute alloyed shell containing both Au and Pd, with a low Pd content of around 10 atom %. By employing the (photo)electrocatalytic nitrite reduction reaction (NO_2_RR) as a model transformation, experimental and theoretical analyses show that this design enables enhanced catalytic activity and selectivity under visible light illumination. We found that the optimized Pd distribution in the alloyed shell allowed for stronger interaction with key adsorbed species, leading to improved catalytic activity and selectivity, both under no illumination and under visible light excitation conditions. The findings provide valuable insights for the rational design of antenna-reactor plasmonic-catalytic NPs with controlled activities and selectivity under visible light irradiation, addressing critical challenges to enable sustainable molecular transformations.

Plasmonic catalysis is a growing field within photocatalysis, enabling one to harvest visible or near-infrared light to accelerate molecular transformations because of the localized surface plasmon resonance (LSPR) excitation in plasmonic nanoparticles (NPs).^1,2^ Plasmonic catalysis has been applied to provide enhanced catalytic activity for reactions such as hydrogenation, reduction, oxidation, and coupling.^3,4^ Yet, to take advantage of the full potential of plasmonic catalysis for enhancing reaction rates, more work is needed to better control the selectivity of these systems.^5−7^ To achieve this control, a better understanding of the factors that lead to changes in the selectivity under LSPR excitation is needed. Tuning the catalytic function of plasmonic nanoparticles is challenging because common plasmonic materials are limited in the number of reactions to which they may demonstrate excellent catalytic activity and selectivity.^5,8,9^ In order to circumvent this problem, hybrid plasmonic-catalytic antenna-reactor NPs have been employed.^10−13^ Here, the plasmonic component harvests energy from visible light through the LSPR (generating excited hot chargers and localized heating), which is harvested or transferred to the catalytic sites to drive a specific molecular transformation.^14−16^

Core−shell, core−satellite, and alloys (Figure 1A−C, respectively) represent the state-of-the-art designs for plasmonic-catalytic antenna-reactor NPs.^10−12^ In the core−shell NPs, the plasmonic component is encased within the shell of the catalytic material. This allows for the isolation of the core from reactants while the shell can be tailored to provide the desired catalytic sites. In the core−satellite NPs, the plasmonic core is not isolated, and smaller NPs of the catalytic component (satellites) are deposited around larger NPs of the plasmonic core. In alloys, the plasmonic and catalytic components are mixed through the NPs. These systems have been extensively employed to enable the study and optimization of catalytic properties.^10−12,14,17−20^ Nevertheless, they often compromise functionality, as the incorporation of the catalytic component into these designs usually leads to a decrease in the optical properties (thereby decreasing the potential plasmonic enhancement of the catalytic activity).

**Figure 1:**
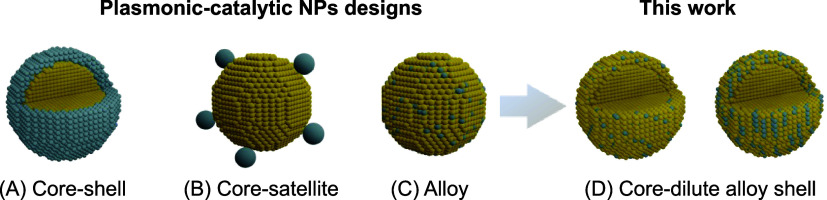
(A−C) Plasmonic-catalytic antenna-reactor NP morphologies: (A) core−shell, (B) core−satellite, and (C) alloyed NPs. The plasmonic and catalytic components are shown in yellow and gray, respectively. (D) NP morphologies developed herein comprise a plasmonic core (Au) and a plasmonic-catalytic alloyed AuPd shell containing low contents of the catalytic metal (Pd, approximately 10 atom %).

To overcome these limitations and simultaneously maximize both optical and catalytic properties, we here demonstrate the synthesis of bimetallic Au@AuPd NPs by focusing on the integration of ultralow loadings of catalytically active palladium into the surface of Au NPs. The goal is to achieve NPs with an Au core and a dilute alloyed AuPd shell with low Pd contents (around 10 atom %). This is illustrated in Figure 1D. Due to the high catalytic activity of Pd for certain reactions, this can allow for applications in plasmonic catalysis and solar-driven chemistry, where both optical and catalytic activities can be optimized. By combining experimental investigations and theoretical analyses, we elucidate how this Au@AuPd NP design enables enhanced catalytic activity and selectivity under visible light illumination to enable their utilization in a wide range of catalytic processes.

## Results and Discussion

The Au@AuPd NPs were prepared by a seeded growth approach using Au NPs as seeds, K_2_PdCl_4_ as a Pd precursor, ascorbic acid as a reducing agent, and water as the solvent at 70 °C. Two samples where the ratio between Au NPs and the Pd precursor was adjusted led to samples denoted Au_97_Pd_3_ and Au_99.7_Pd_0.3_, respectively, based on the microwave plasma atomic emission spectroscopy (MP-AES) elemental analysis (Table S1). Here, a surface alloy containing Au and Pd forms when the reduced Pd species interacts with the Au-based surface.

**Figure 2:**
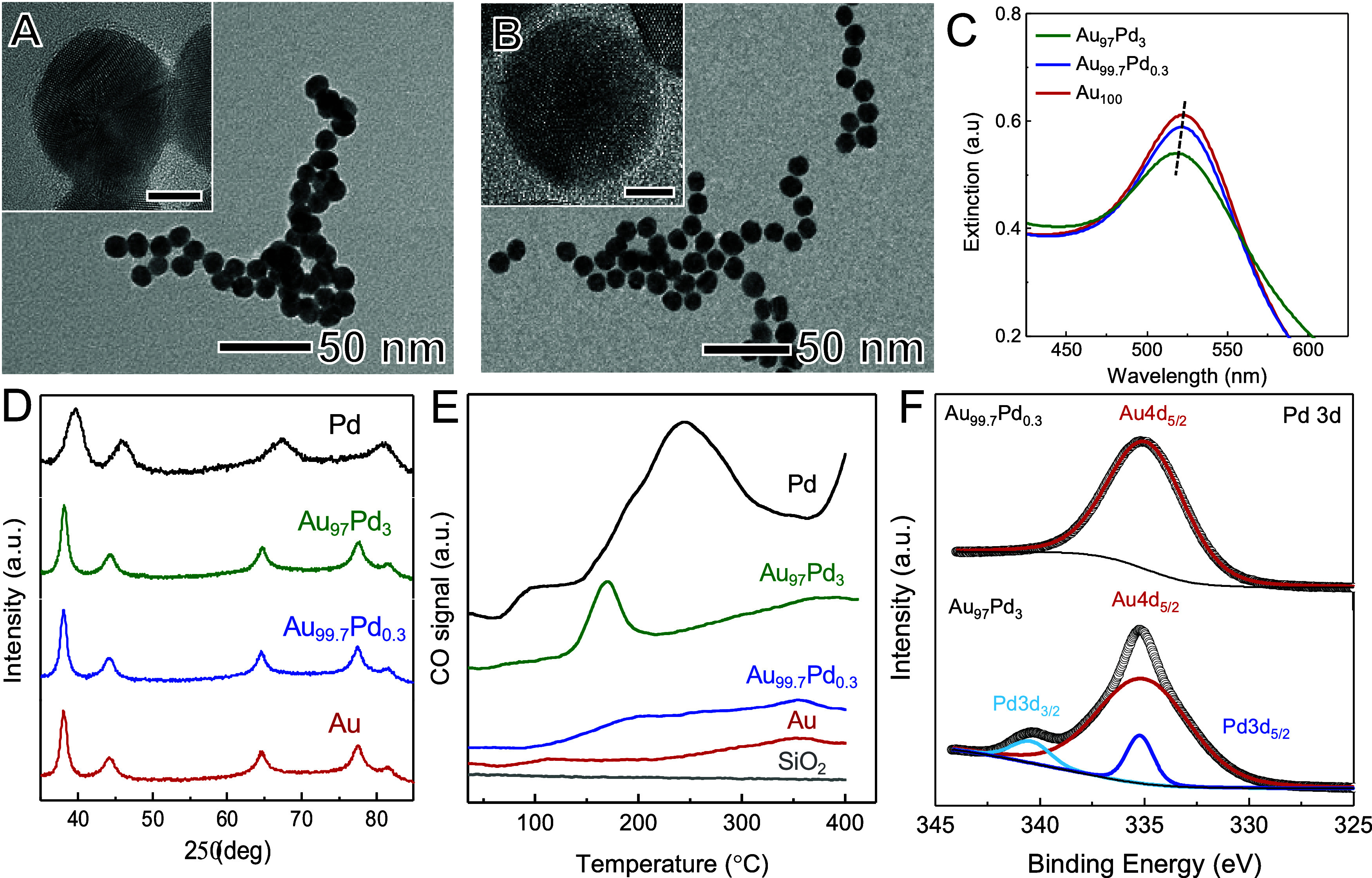
(A, B) TEM images of Au_99.7_Pd_0.3_ and Au_97_Pd_3_ NPs. Scale bars in the insets correspond to 5 nm. (C) UV−vis extinction spectra were recorded from aqueous suspensions containing the Au_99.7_Pd_0.3_ (blue trace) and Au_97_Pd_3_ (green trace) NPs. The spectrum of Au NPs employed as seeds during the synthesis is also shown for comparison (red trace). (D) XRD patterns and (E) CO-TPD for Au_99.7_Pd_0.3_ (blue trace) and Au_97_Pd_3_ (green trace) NPs. The XRD and CO-TPD data for Au and Pd NPs and SiO_2_ (CO-TPD only) are also shown for comparison (red and black traces, respectively).

Figure 2A,B shows transmission electron microscopy (TEM) images of Au_99.7_Pd_0.3_ and Au_97_Pd_3_ NPs. The particles are spherical and relatively uniform in size, with diameters of 15.4 ± 1.4 and 16.2 ± 1.7 nm for Au_99.7_Pd_0.3_ and Au_97_Pd_3_, respectively (Figure S1). These diameters are only slightly larger than the original Au seed particles, which had identical morphology and diameters of 14.4 ± 1.7 nm (Figure S2), as expected from the low Pd content in the added shells. The UV−VIS extinction spectra (Figure 2C) from aqueous suspensions of Au_97_Pd_3_, Au_99.7_Pd_0.3_, and Au NPs (green, blue, and red traces, respectively) show that all NPs exhibit extinction bands within the visible spectrum. This is due to the excitation of a dipolar LSPR mode, in which a gradual reduction in the intensity of the band and a slight blue shift is seen when the Pd content in the NPs increases.^21^ The LSPR bands are centered at 517, 520, and 522 nm for Au_97_Pd_3_, Au_99.7_Pd_0.3_, and Au NPs, respectively. These variations in intensity and position are consistent with the deposition of Pd on Au and indicate that lower Pd loadings result in more pronounced LSPR bands.^21^ The powder X-ray diffraction (XRD) diffraction patterns (Figure 2D) acquired from the Au_99.7_Pd_0.3_ and Au_97_Pd_3_ NPs supported on SiO_2_ substrates (blue and green traces, respectively) only show peaks which are assigned to *fcc* Au (the XRD patterns for Au and Pd NP references are also shown as red and black traces, respectively). This agrees with the low Pd content in the samples and shows that no crystalline impurities were present at detectable amounts. The Au_99.7_Pd_0.3_ and Au_97_Pd_3_ NPs have a low Pd content and a core−shell structure with a thin (1.4 and 1.5 nm) alloyed AuPd shell (according to electron microscopy results discussed below). Given the low Pd content in the NPs and the size of the alloyed shell, shifts to higher angles in the XRD peaks of Au due to Pd incorporation are expected to be very subtle. Moreover, due to the core−shell morphology, the dominant diffraction signal still arises from the Au core, which can mask subtle shifts in the shell’s diffraction peaks. These observations are in agreement with our results in which a clear slight shift to higher angles due to the presence of Pd was not obvious.

CO temperature-programmed desorption (CO-TPD) was performed to gain insights into the surface properties of the NPs. CO-TPD is an important method to determine CO-metal bond energy in Pd and can be described by including electrostatic interactions and π-back bonding from the Pd to CO.^22,23^Figure 2E shows the CO-TPD profiles for the Au_99.7_Pd_0.3_ and Au_97_Pd_3_ NPs supported on SiO_2_ (blue and green traces, respectively). Au and Pd NP references supported on SiO_2_ and the uncoated SiO_2_ support are also shown as references (red, black, and gray traces, respectively). The CO-TPD profile for Pd NPs displays distinctive peaks at 100 and 250 °C, which can be assigned to weakly and moderately adsorbed CO species, respectively.^22^ It has been proposed that weakly adsorbed CO can correspond to CO bound to terrace atop Pd sites, while moderately adsorbed CO is bound to bridge and edge sites of Pd as well as to 3-fold hollow sites.^24,25^ For the Au@AuPd NPs, the intensities of the CO desorption features decrease with a decreasing Pd content. In fact, only a broad and weak signal centered at 190 °C is detected for Au_99.7_Pd_0.3_ NPs (blue trace). For Au_97_Pd_3_ NPs, the desorption signal is shifted to 170 °C (relative to 250 °C in Pd). This shift to lower temperatures for Au_97_Pd_3_ and Au_99.7_Pd_0.3_ NPs relative to that of pure Pd agrees with the weakening of the CO adsorption as alloying with Au takes place.^22,24−26^ Interestingly, the higher temperature peak for Au_99.7_Pd_0.3_ compared to Au_97_Pd_3_ indicates that the strength of CO adsorption is higher on Au_99.7_Pd_0.3_ relative to Au_97_Pd_3_ NPs. This peak has a lower intensity in Au_99.7_Pd_0.3_ than in Au_97_Pd_3_ NPs due to the lower surface Pd content. It is plausible that the lower Pd concentration in Au_99.7_Pd_0.3_ leads to differences in the Pd distribution at the surface relative to Au_97_Pd_3_, which could cause a stronger interaction with CO adsorbed on the atop sites. The calculated amount of adsorbed CO corresponds to 0.731 and 0.199 μmol/g_cat_ for Au_97_Pd_3_ and Au_99.7_Pd_0.3_ NPs, respectively. It is important to note that CO does not adsorb on Au or SiO_2_ under our conditions, as illustrated by the absence of any signals in the CO-TPD profiles for Au and SiO_2_.

The X-ray photoelectron spectroscopy (XPS) spectra for the Au 4f core level for Au_97_Pd_3_ and Au_99.7_Pd_0.3_ NPs are shown in Figure S3. Two peaks at 88.0 and 84.0 eV, assigned to the Au 4f_7/2_ and 4f_5/2_ doublets of Au^0^, were detected for both samples.^27^ No shifts in the Au peaks were detected when the Pd content was varied, which can be attributed to the low Pd loading in the samples or the absence of detectable electronic interactions between Au and Pd in our samples (the interaction would be expected in an alloy with a higher Pd loading). Figure 2F shows the XPS spectra for Au_97_Pd_3_ and Au_99.7_Pd_0.3_ NPs in the Pd 3d core-level region. Usually, Pd^0^ is characterized by the presence of a doublet with peaks at 335 and 340 eV assigned to 3d_5/2_ and 3d_3/2_, respectively.^28^ These signals can be identified in the Au_97_Pd_3_ NPs (bottom trace). Our data also shows that the Pd 3d_5/2_ signal overlaps with and is expected to be overshadowed by the Au 4d_5/2_ peak at 335 eV because of the low Pd content in this sample.^29^ Thus, the Pd 3d_3/2_ peak more clearly shows the presence of Pd. From the Au_99.7_Pd_0.3_ sample, no detectable Pd signals were observed because of the low Pd content.

**Figure 3:**
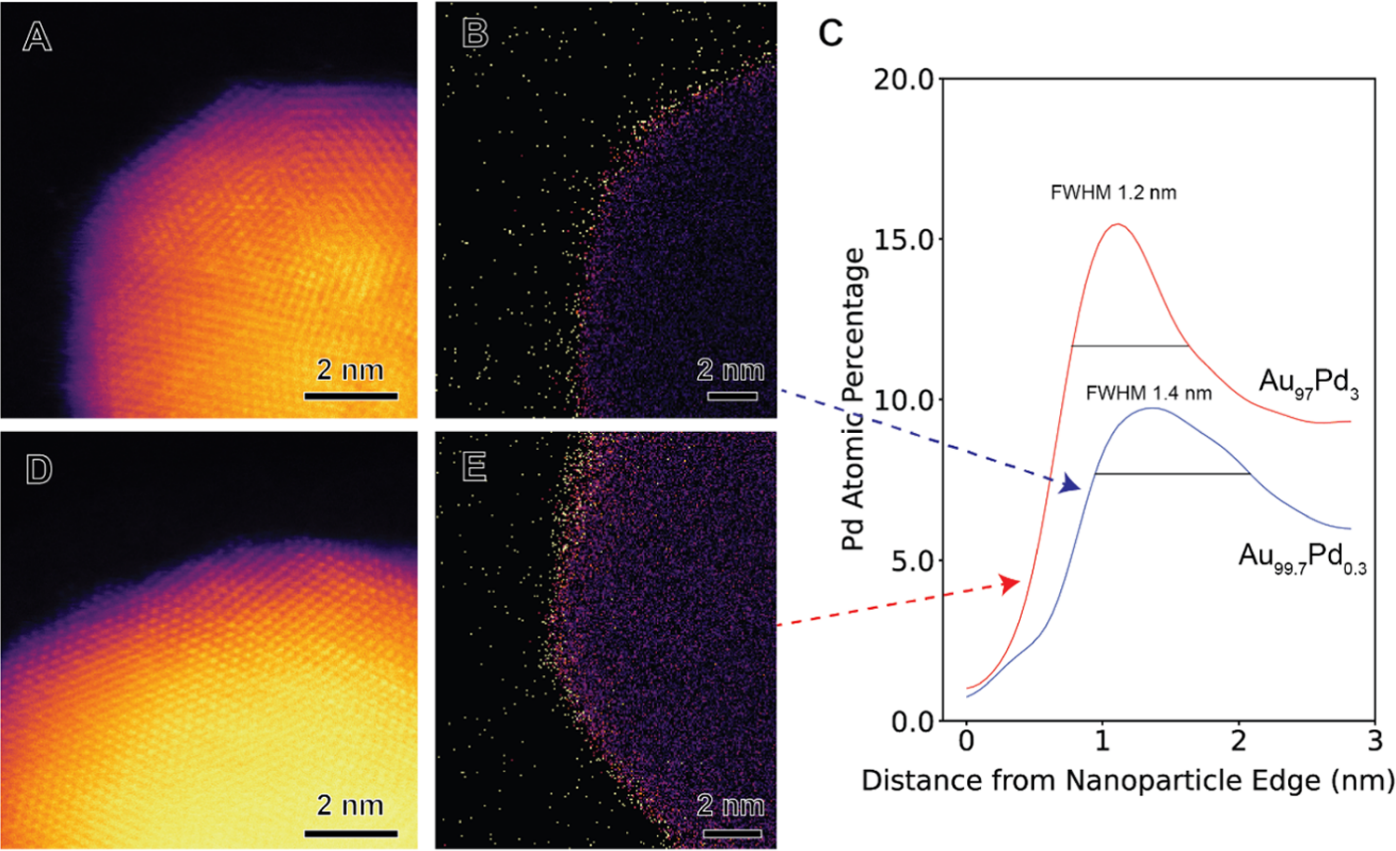
Elemental distributions in Au_99.7_Pd_0.3_ and Au_97_Pd_3_ NPs. (A, D) STEM−HAADF images, (B, E) STEM−EDX elemental maps for Pd in Au_99.7_Pd_0.3_ (A, B) and Au_97_Pd_3_ NPs (D, E). (C) EDX line profiles averaged over pixels certain distances from the NP edges for Au_99.7_Pd_0.3_ and Au_97_Pd_3_ NPs from the NPs in (B) and (E), respectively. The full width at half-maximum (FWHM) of the Pd surface enrichment peak is indicated in (C) for both samples.

Figure 3A−F shows high-angle annular dark field (HAADF) scanning transmission electron microscope (STEM) images, STEM energy-dispersive X-ray fluorescence (STEM−EDX) elemental maps, and EDX elemental line scans for Au_99.7_Pd_0.3_ and Au_97_Pd_3_ NPs. The imaging at atomic resolution of both Au_99.7_Pd_0.3_ and Au_97_Pd_3_ NPs (Figure 3A,D, respectively) indicates that Pd is not present as protruding surface islands, but the NPs have relatively smooth surfaces. STEM-EDX analysis shows the distribution of Pd at the surface of the NPs for both Au_99.7_Pd_0.3_ and Au_97_Pd_3_ (as depicted in Figure 3B,E, respectively). The HAADF STEM intensity line scans show a smoothly decaying surface profile, consistent with the alloying of Au and Pd at the surface rather than a pure Pd shell (Figure S4). This is also supported by the STEM-EDX line scans (Figure S9) that show the Au signal persisting to the edge of the nanoparticle in both cases. Extracting averaged elemental profiles based on the distance of pixels from NP edges reveals similar Pd-rich AuPd shell thicknesses of 1.4 nm for Au_99.7_Pd_0.3_ and 1.2 nm for Au_97_Pd_3_ (Figure 3C), corresponding to a thickness of ∼4 and ∼5 atomic layers, respectively. The elemental quantification of STEM-EDX is challenging for the low alloying contents but was achieved by Python-based fitting using all of the available X-ray peaks for each element (see the Supporting Information for full details). Analysis of the summed STEM-EDX spectra for multiple NPs (as depicted in Figure S5) provided mean compositions of 92.7 atom % Au and 7.2 atom % Pd for Au_97_Pd_3_ NPs, and 96.3 atom % Au and 3.7 atom % Pd for Au_99.7_Pt_0.3_ NPs with standard deviation errors of 0.5 and 0.4 atom % (Figures S6 and S7). However, as Pd was only found to be present within the shell region, a better estimate of the NP elemental content is achieved by assuming a core@shell NP morphology with the shell thickness given by the full width at half-maximum (FWHM) of the measured Pd enrichment at the surface (see Figure S8). This allows estimation of the AuPd shell compositions as 25.3 atom % Pd and 10.2 atom % Pd for Au_97_Pd_3_ and Au_99.7_Pd_0.3_, respectively. These results indicate the formation of a Au core and a AuPd alloyed shell in both the Au_99.7_Pd_0.3_ and Au_97_Pd_3_ NPs, where the concentration of Pd in the alloyed shells decreases as the loading of Pd in the NPs decreases.

**Figure 4:**
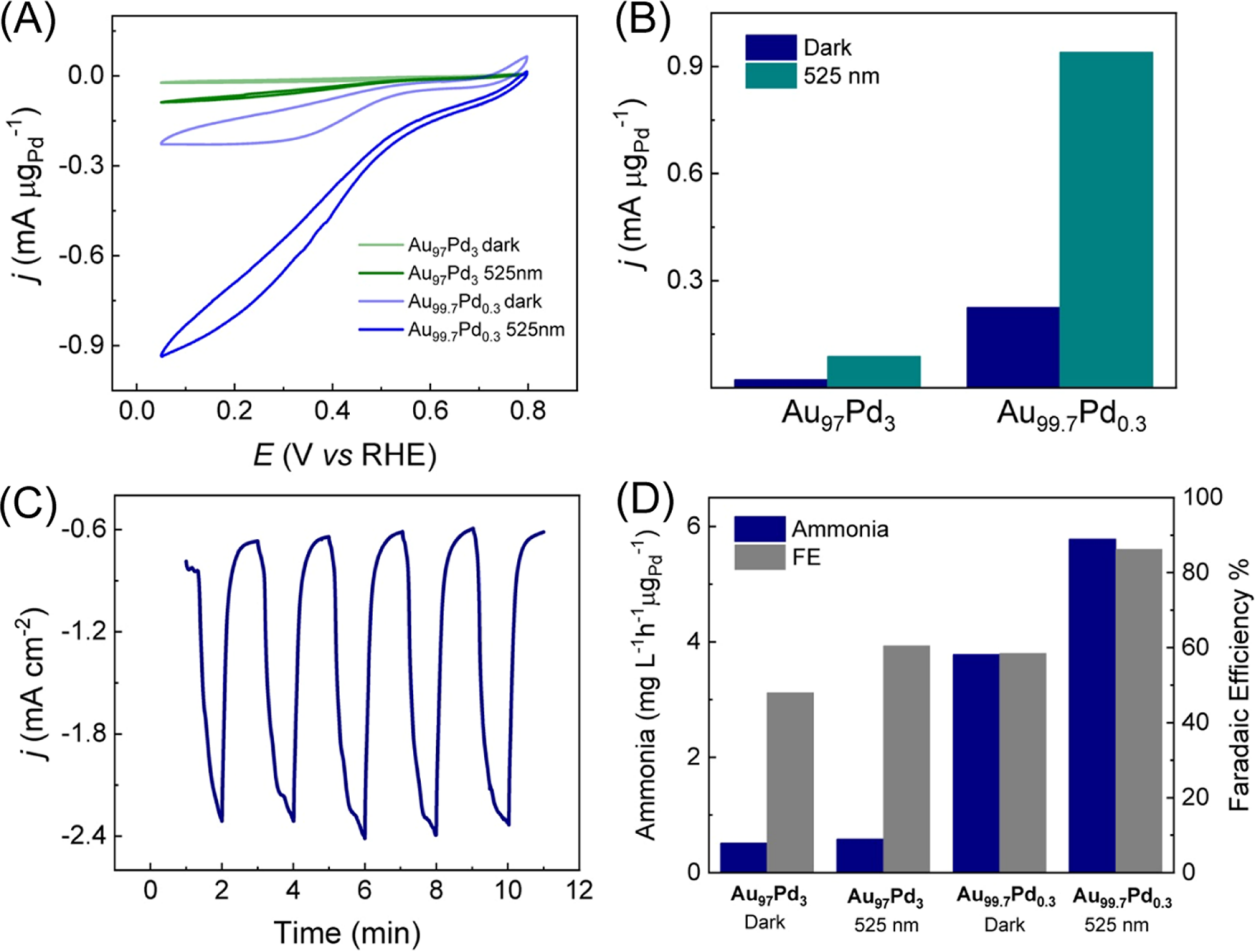
(A) Cyclic voltammograms recorded at 0.010 V s^−1^ for Au_97_Pd_3_ (green trace) and Au_99.7_Pd_0.3_ (blue trace) in the presence of 10 mmol L^−1^ NaNO_2_ in 0.1 M HClO_4_. Measurements in the dark (lighter line) and under light irradiation (525 nm, darker line) are shown. (B) Bar graph depicting the current density at 0.05 V for Au_99.7_Pd_0.3_ and Au_97_Pd_3_ under the dark (blue column) and light irradiation (green column) conditions. (C) Chronoamperometric curves for Au_99.7_Pd_0.7_ NPs were recorded at 0.050 V in 0.1 M HClO_4_ containing 10 mmol L^−1^ NaNO_2_ in on/off conditions under 525 nm light irradiation. (D) Ammonia concentration (blue bar) and Faradaic efficiency to/of ammonia production (gray bar) at −0.1 V (RHE) on Au_99.7_Pd_0.3_ and Au_97_Pd_3_ NPs in the dark and under 525 nm light irradiation conditions.

We then investigated how the Au@AuPd core@shell morphology, the different compositions of the shells, and the LSPR excitation in the visible range influence the electrocatalytic activities of the Au_99.7_Pd_0.3_ and Au_97_Pd_3_ NPs. We employed nitrite (NO_2_^−^ reduction reaction (NO_2_RR)) as a model transformation (as shown in Figure 4). The cyclic voltammograms obtained in the presence of NO_2_^−^ exhibited characteristic features of the reduction of NO_*x*_ compounds (Figures 4A and S10, respectively).^30^ Voltammograms obtained for Au NPs in the presence of NO_2_^−^ and for Au_99.7_Pd_0.3_ and Au_97_Pd_3_ NPs under blank conditions (in the absence of NO_2_^−^) can be found in Figure S11, showing that the samples have negligible (photo)electrochemical activity under these conditions. The current densities for both Au_99.7_Pd_0.3_ and Au_97_Pd_3_ samples increased under visible light illumination because of the LSPR-enhanced NO_2_RR. Figure 4A shows cyclic voltammograms where the current densities are normalized by the mass of Pd to better compare the performance of the samples, as Pd is the only electrocatalytically active species under our employed conditions. These indicate that the current densities for the reduction of NOx compounds are higher both in the dark and under light illumination conditions for the Au_99.7_Pd_0.3_ NPs compared to those of Au_97_Pd_3_. The calculated mass activities at 0.05 V are depicted in Figure 4B. For both Au_99.7_Pd_0.3_ and Au_97_Pd_3_ NPs, activities increased under plasmonic excitation relative to those under dark conditions. Specifically, a 4-fold increase was detected in both samples (from −0.23 to −0.94 mA μg_Pd_^−1^ in Au_99.7_Pd_0.3_ and from −0.023 to −0.089 mA μg_Pd_^−1^ in Au_97_Pd_3_). It can also be observed that Au_99.7_Pd_0.3_ NPs displayed higher activities compared to Au_97_Pd_3_ under both dark and light irradiation conditions. Specifically, 10-fold and 10.6-fold increases in mass activity were detected in the dark and light illumination conditions, respectively. This indicates that the more dilute Pd distribution and lower Pd content in the Au_99.7_Pd_0.3_ surface-alloyed shell were important for the increased catalytic activity even without LSPR excitation.

Under dark conditions, the plasmonic effects are absent, and the catalytic activity primarily depends on the intrinsic properties of the Pd within the AuPd shell. For both Pd concentrations investigated in this study, the thickness of the alloyed surface AuPd shell is similar, and the difference relies mainly on the Pd concentration, which leads to Pd being more dilute in terms of its distribution in the shell having a lower Pd content. This more dilute distribution enhances the interaction between Pd sites and the reactants involved in the NO_2_RR, promoting selective adsorption and activation of intermediates crucial for the formation of NH_3_. In contrast, a higher Pd loading can lead to the formation of more Pd-rich regions, which may exhibit different electronic properties and potentially introduce competing pathways or side reactions that reduce selectivity toward NH_3_. Furthermore, the electronic interaction between Au and Pd in the dilute alloy plays a significant role, as also corroborated by our DFT calculations (discussed later in the text). The alloying effect at low Pd content can modify the electronic structure of Pd, making it more effective for the NO_2_RR even without plasmonic enhancement by enhancing the binding and activation of nitrogen-containing intermediates, leading to a higher yield of NH_3_. Under plasmonic excitation, the increase in catalytic activity for the Au_99.7_Pd_0.3_ sample can also be related to the higher light absorption enabled by the lower Pd content. The on−off transients for Au_99.7_Pd_0.3_ NPs under chopped light excitation at 525 nm (Figure 4C) show fast and reproducible current responses to the on−off illumination cycles in agreement with the NO_2_RR plasmonic enhancement during/under light illumination conditions via hot charges and localized heating.^31^

Next, we investigated how the compositional features of the NPs and plasmonic effects influence the reaction selectivity toward the formation of NH_3_. As shown in Figure S12, the NO_2_RR can lead to a variety of products, including NO, N_2_, N_2_O, N_2_OH, and NH_3_. Under acidic conditions employed herein, it is reported that NO_2_^−^ can be easily converted into NO_(ad)_ at the surface of the catalyst, which represents the key intermediate for the NO_2_RR.^32^ In order to evaluate the reaction selectivity under dark and light conditions, we quantified the amount of NH_3_ produced by the indophenol method (Figure S13).^33^Figure 4D shows the concentrations of NH_3_ produced (blue bars) under dark and light irradiation conditions for Au_99.7_Pd_0.3_ and Au_97_Pd_3_ NPs together with the respective Faradaic efficiency percentages (FE, gray bars). The amount of NH_3_, and thus the selectivity toward NH_3_, was significantly higher for the Au_99.7_Pd_0.3_ relative to Au_97_Pd_3_. Moreover, with Au_99.7_Pd_0.3_, a significant increase in selectivity was detected under the light irradiation conditions, showing that the plasmonic excitation not only increases reaction rates (catalytic activity) but also enables control over reaction selectivity. The detected NH_3_ concentrations after 1 h of electrolysis at 0.05 V for Au_97_Pd_3_ NPs were 0.5 and 0.6 mg L^−1^ h^−1^ μg_Pd_^−1^ under dark and light excitation conditions, respectively. This corresponded to FE values of 48 and 61%, respectively. On the other hand, the NH_3_ concentration was 3.8 and 5.8 mg L^−1^ h^−1^ μg_Pd_^−1^ under the dark and light excitation conditions, respectively, for the Au_99.7_Pd_0.3_ NPs. This corresponds to an increase of 7.6 and 9.7-fold for the light and dark conditions, respectively, relative to those of the Au_97_Pd_3_ NPs, and a significant enhancement in selectivity for the formation of the desirable NH_3_ due to the plasmonic excitation. The FEs were also improved for Au_99.7_Pd_0.3_ relative to Au_97_Pd_3_ to 58.5 and 86.2% under dark and light excitation conditions, respectively.

Upon light absorption, the excitation of localized surface plasmon resonance (LSPR) leads to strong electric field enhancements (*E*/*E*_0_) near the surfaces of plasmonic NPs.^1^Figure S14 shows DDA simulations on the magnitudes and special distribution of the electric field enhancements for Au_99.7_Pd_0.3_ and Au_97_Pd_3_ NPs excited at 525 nm. It can be observed that both NPs display similar electric field enhancements, and the fields are strongly concentrated along the polarization direction of the incoming electromagnetic wave. The LSPR excitation primarily undergoes nonradiative Landau damping to produce energetic charge carriers that quickly redistribute their energy, generating hot electrons and hot holes with a quasi-Fermi−Dirac distribution.^34,35^ These electron−hole pairs further relax by transferring energy to the phonon modes of the metal nanoparticles, resulting in localized heating effects.

In terms of increasing reaction rates, both energetic hot carriers and localized heating induced by LSPR can contribute to plasmonic catalysis.^36,37^ However, the untangling of the effect of localized heating and hot charge carriers over activities remains challenging.^4,38^ In plasmonic electrocatalysis, it has been reported that the local temperature at the electrocatalyst/medium interface under laser irradiation from 0 to 2.55 W/cm^2^ (more intense than our LED source) was only moderately higher than that under dark conditions and that the temperature increase resulting from photothermal heating has low influence on the HER performances.^31^ Also, we tried to mitigate the heat effects herein by performing all of the experiments in a temperature-controlled system. Figure S15 suggests a mechanism for the enhanced NO_2_RR performances under light illumination based on the generation of LSPR-excited charge carriers. LSPR excitation leads to the generation of hot carriers from the Au plasmonic core. The hot electrons generated by the Au NPs can transfer to the AuPd shells, where they participate in enhancing reaction rates by activating surface adsorbates.^39,40^ The holes are transported to the counter electrode with the assistance of an external voltage. This activation mechanism is supported by the detected linear dependence of current density with light intensity (Figure S16).^38,41^

Beyond increasing reaction rates, plasmonic excitation also leads to a control over reaction selectivity.^7,42,43^ This can be achieved through several pathways, including plasmon-mediated selective adsorption, plasmon-mediated selective activation, and plasmon-mediated selective desorption.^43^ In plasmon-mediated selective adsorption, the electromagnetic field due to the LSPR can add an optical force to selectively concentrate polar molecules on the catalyst surface and change the adsorption energy.^43^ When several kinds of reactants with different functional groups are involved in reactions, the molecules with larger dipole moments tend to be concentrated near the plasmonic catalyst with the assistance of a local electromagnetic field, resulting in the preferential adsorption and activation of such molecules. In plasmon-mediated selective activation, LSPR-excited charge carriers generated by plasmonic excitation can selectively populate specific electronic states of adsorbed molecules, favoring certain reaction pathways over others.^44,45^ For example, the injection of hot electrons into the antibonding molecular orbitals enables a reduced bond order, making the breakage of chemical bonds easier. Due to the LSPR, the energy of highly energetic hot electrons can be tailored to allow for their specific injection into particular adsorbate orbitals. Such adsorbates are thus activated and converted preferentially. Finally, in terms of plasmon-mediated selective desorption, the desorption of intermediates and products is sometimes the rate-limiting step of chemical reactions. In plasmonic catalysis, hot carrier injection to adsorbates may lead to the vibrational excitation of adsorbates, which weakens the metal−adsorbate interaction and promotes the desorption of adsorbates from the catalyst surface, which alters the surface coverage of intermediate species, which potentially results in a modified reaction pathway.^10,42^

It is important to note that it has been reported that Pd NPs can display LSPR excitation in the visible range via control of size (when NPs are above 50 nm) or shape (cubes, cages, flowers, stars, and plates, for example).^46^ However, below 20 nm, spherical Pd NPs display an LSPR in the UV region. Therefore, in our Au_99.7_Pd_0.3_ and Au_97_Pd_3_ NPs, it is expected that the LSPR excitation from Pd plays no significant role in the detected plasmonic-catalytic activities.

**Figure 5:**
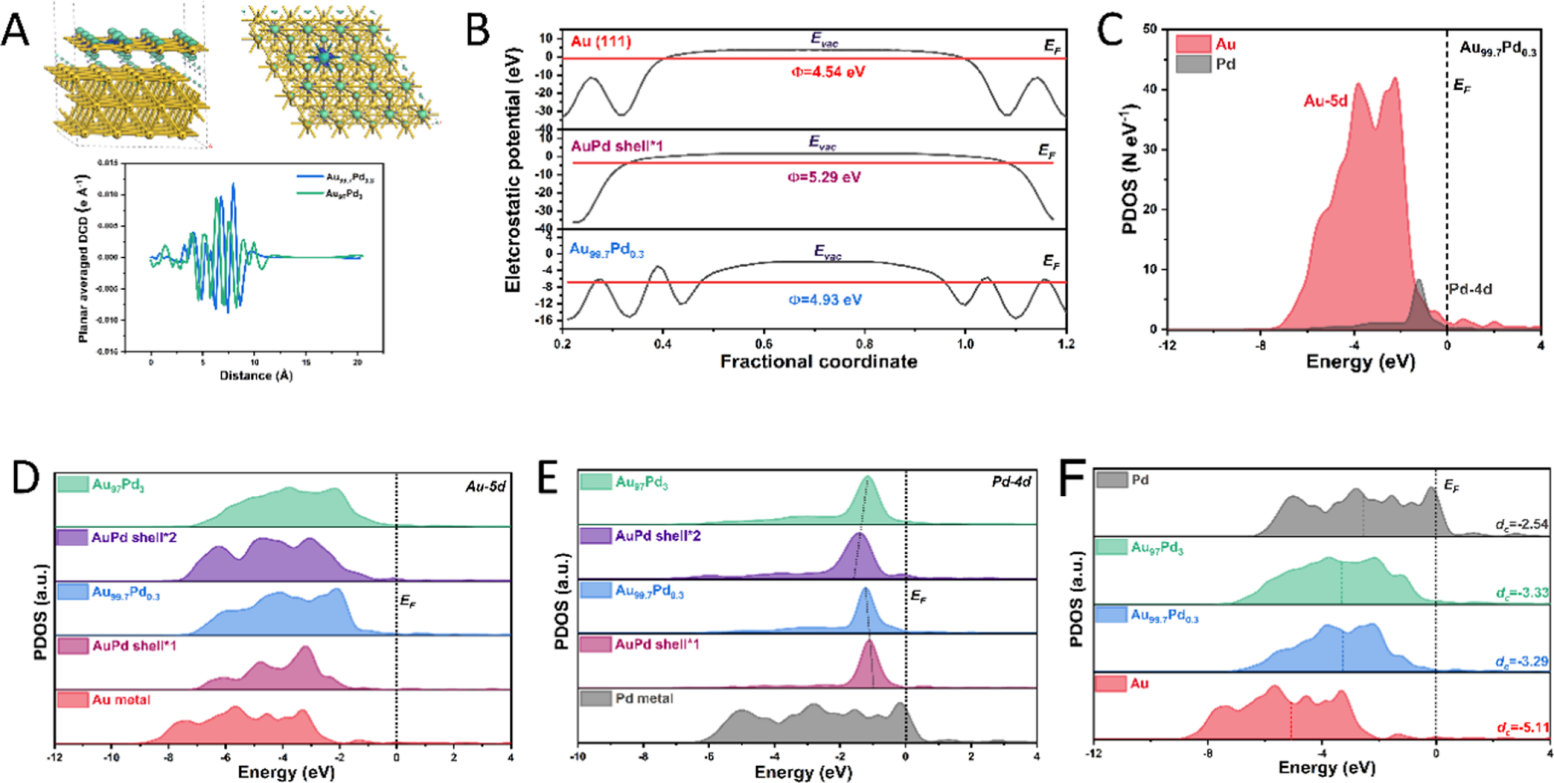
(A) Charge density differences in the constructed Au_99.7_Pd_0.3_ NP model (side and top views) and the plane-averaged differential charge density (DCD) across the interface (bottom panel). The blue and bright green contours represent the regions of electron accumulation and depletion, respectively. (B) Electrostatic potentials for Au (111), AuPd alloy shell, and Au_99.7_Pd_0.3_ models. (C) Projected density of state (PDOS) curves for Au_99.7_Pd_0.3_ model NPs. PDOS of (D) Au-5d and (E) Pd-4d within different coordination environments. (F) d-PDOS on Au, Au_99.7_Pd_0.3_, Au_97_Pd_3_, and Pd models. Shell*1 and shell*2 refer to the AuPd alloy shell in Au_99.7_Pd_0.3_ and Au_97_Pd_3_ NPs, respectively.

We performed density functional theory (DFT) calculations to gain a further understanding of the observed catalytic activity and selectivity enhancement. The theoretical models used to understand the electronic structure of the Au_99.7_Pd_0.3_ and Au_97_Pd_3_ NPs are depicted in Figure S17. Charge density difference (CDD, Figure 5A) and Mulliken charge (Figure S18 and Table S2) analyses show the electron-rich nature of the Pd site and the local charge redistribution at the surface. In addition, electron localization was evaluated by electron local function (ELF) to show the bond characteristics and charge transfer between Pd and the surrounding Au atoms.^47^ As shown in Figure S19, the ELF value around the Pd atom in the Au_99.7_Pd_0.3_ model was lower than that around the Au atoms, indicating that the electrons surrounding the Pd atom are more delocalized.^48^ The observed local charge redistribution and charge density regions around the isolated Pd atoms at the surface in the Au_99.7_Pd_0.3_ model may be responsible for favoring the migration of the LSPR-induced hot charges to the Pd sites under the light irradiation, leading to the higher reaction rates. As for the Au_97_Pd_3_ NPs, the larger number of Pd atoms means that the local charge distribution mainly occurs at the Au-AuPd interface, not on the surface (Figure S20). The planar average charge density differences (Figure 5A, bottom panel) show the formation of an internal electric field within the core−shell structure for both Au_99.7_Pd_0.3_ and Au_97_Pd_3_ NP models. This is also supported by the calculated work functions (WF) of pure Au, the AuPd shell, and Au_99.7_Pd_0.3_ (Figure 5B). Due to the difference in the WF values between pure Au (4.54 eV) and the AuPd shell (5.29 eV), the contact between the Au core and the AuPd shell could cause charge redistribution across the interface, enabling electron transfer from Au to AuPd until their work functions become equivalent/aligned (for Au_99.7_Pd_0.3_, WF is 4.93 eV).^49^ Therefore, under the light excitation conditions, LSPR-excited hot electrons would spontaneously migrate to the AuPd shell, while holes are transferred to the counter electrode, contributing to the enhanced performance under the LSPR excitation by boosting separation of the photon-generated carriers.^50^

**Figure 6:**
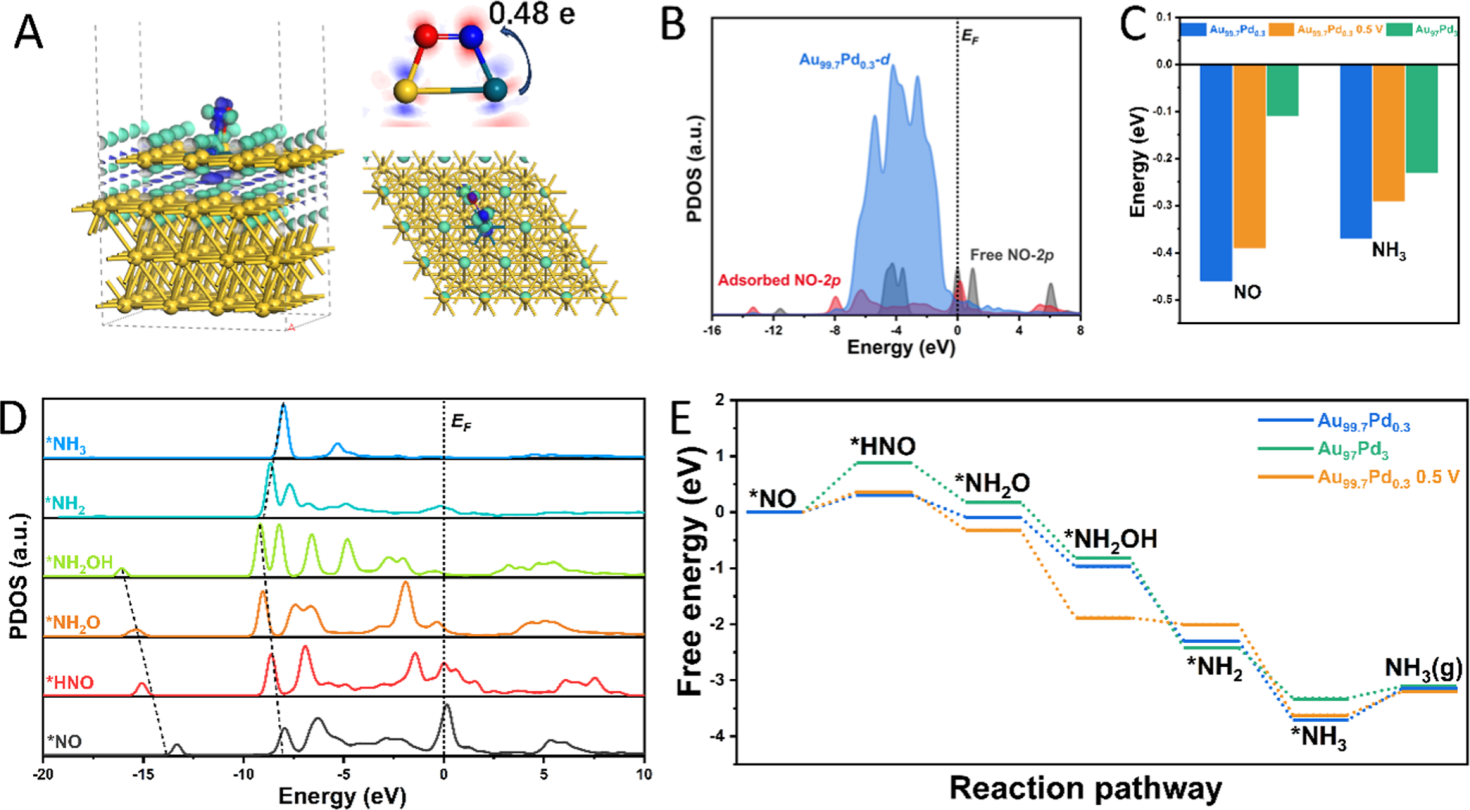
(A) Charge density differences and Mulliken charge analysis in the constructed Au_99.7_Pd_0.3_ NP model containing a NO molecule adsorbed at the proposed surface sites. The blue and bright green contours represent the regions of electron accumulation and depletion, respectively. For two-dimensional (2D) maps, the scale from blue to red is −0.4 to 0.4 e. (B) PDOS for a free (gray) and absorbed (red) NO molecule (2p levels) at the surface site in the Au_99.7_Pd_0.3_ NP model. (C) Adsorption energies of NO and NH_3_ on Au_99.7_Pd_0.3_ (with/without applied voltage) and Au_97_Pd_3_ (111) model NP surfaces. (D) PDOS of key intermediates in Au_99.7_Pd_0.3_ NP model during the reaction progress. (E) Free energy profiles for the reaction pathway on Au_99.7_Pd_0.3_ (with/without applied voltage) and Au_97_Pd_3_ models.

The projected partial density of states (PDOS) shown in Figure 5C indicates that the Pd-4d orbital is located close to the Femi level (*E*_F_, band center at −1.56 eV), while the Au-5d orbital is buried at a deeper position away from *E*_F_ (band center at −3.48 eV). This supports the assertion that Pd acts as the main active site for the catalytic reaction due to the stronger interaction with adsorbed NO molecules (Figure 6B) and that, under the LSPR excitation conditions, hot electrons transferred to the Pd sites can contribute to the accelerated reaction rates.^51^ The site-dependent PDOS of the Au-5d bands was also calculated (Figure S21). From the bulk to the Au-AuPd interface and then into the AuPd shell (from bottom to top in Figure S21), the Au-5d PDOS exhibited a trend toward lower energy (further to the Fermi energy). For a more in-depth understanding of the electronic structures, we compared the electronic structures of Au-5d and Pd-4d orbitals in different environments (Figure 5D,E). For Au_99.7_Pd_0.3_ and Au_97_Pd_3_ models, both the Au-5d bands exhibited an upshifting trend compared with those of pure Au metal and individual shells consisting of AuPd alloys. This indicates an improved electroactivity and effective d−d orbital coupling within the obtained core−shell structure relative to those of the pure metals or alloy counterparts. On the other hand, both Au_99.7_Pd_0.3_ and Au_97_Pd_3_ displayed more positive positions of the overall Pd-4d band relative to the d-band center of the Pd metal (Figure 5E). However, the Pd-4d band in Au_99.7_Pd_0.3_ was negatively shifted compared with that in the AuPd shell (shell*1), which is contrary to the Au_97_Pd_3_ behavior. This might be caused by the lower concentration of Pd, which can optimize the binding strengths between Pd active sites and intermediates.^52^ This is consistent with our results from CO-TPD. The overall electronic structure comparison with the pure Au metal also indicated that the overall d-band centers (*d*_c_) have an increased density of states after the introduction of the AuPd alloy shell (Figure 5F). This leads to more adsorbate antibonding states being pulled above the *E*_F_ and enhancing the electron transfer between the adsorbate and active sites.^53^ Therefore, these results support our experimental findings on the improved electrocatalytic activity for Au_99.7_Pd_0.3_ NPs relative to that of Au_97_Pd_3_ under dark and light irradiation conditions.

Figure S22 shows the calculated reaction pathway for the reduction of NO to NH_3_. Figure 6A shows the optimized configuration for a NO molecule adsorbed on Pd sites on the Au_99.7_Pd_0.3_ surface. The calculated CDD results suggest that NO adsorption on the Au_99.7_Pd_0.3_ surface results in a stronger local charge redistribution over the active sites than that on the Au_97_Pd_3_ surface (Figure S23), causing more electron migration from the surface to the adsorbed *NO species (supported by quantitative Mulliken charge analysis). The PDOS for adsorbed (*NO) and free NO is shown in Figure 6B and reveals the strong interaction at the surface with the downshift of 2p orbitals upon adsorption.^54^ To quantitatively evaluate the interaction between different Pd sites (Au_99.7_Pd_0.3_ and Au_97_Pd_3_ NPs) and *NO, the projected crystal orbital Hamilton population (pCOHP) of the Pd−N bond (Pd in the surface site and *N, Figure S24) was calculated. The integral COHP value (ICOHP) could be calculated by integrating the partial COHP below the *E*_F_, suggesting the number of bonded electrons between the selected Pd and N atoms and the corresponding bonding strength.^55^ It is notable that the ICOHP of Pd−N in Au_99.7_Pd_0.3_ is larger than that of Au_97_Pd_3_ and demonstrates a stronger NO adsorption (Figure S25). The calculated adsorption energies of NO (Figure 6C) agree with these results, suggesting that control over the composition of Pd in the AuPd shells modulates the adsorption strength of NO at the surface.

The PDOS analysis of NO, the reaction intermediates, and NH_3_ at the Au_99.7_Pd_0.3_ NP surface were calculated (as shown in Figure 6D). With the progress of the hydrogenation reaction, the conversion of *NO to *NH_3_ exhibits two linear-like trends, which might be ascribed to (N−O)σ and (N−H)σ binding energies.^51^ These two linear trends for (N−O)σ and (N−H)σ indicate the substantial efficiency of p–d electron transfer during the NO_2_RR on the Au_99.7_Pd_0.3_ NPs. The energy profiles for the NO_2_RR are shown in Figure 6E. For Au_97_Pd_3_ NPs, the first step (protonation of *NO to *NHO) is identified as the rate-determining step (RDS) and exhibits a large energy barrier of 0.88 eV, which is consistent with the adsorption energy results (Figure 6C). On the other hand, the energy barrier is reduced to 0.31 eV on Au_99.7_Pd_0.3_ NPs due to the optimized electronic properties as a result of the change in surface composition, as described herein. Finally, we performed calculations on the superficial charge distribution of the Au_99.7_Pd_0.3_ NP model in the presence of an applied electric field (0.5 V Å^−1^) to simulate the effect of the LSPR excitation. As shown in Figure S26 and Table S3, the charge distribution at the top surface was altered, and the adsorption energies of NO and NH_3_ were affected (Figure 6C, a slight decrease relative to no applied electric field).

Our results indicate that a more dilute concentration of Pd in the shells can contribute to light adsorption, facilitating the transfer of hot carriers to the Pd sites. This effect can lead to enhanced catalytic activity and increased selectivity toward NH_3_ formation under light excitation, which is an indicator of a larger extent of reduction of NO_2_^−^ than other products. Furthermore, a more dilute concentration of Pd could promote the hydrogenation pathway while decreasing the formation of N_2_, which requires the coupling between two adjacent N-containing species.

## Conclusions

We have developed herein antenna-reactor plasmonic nanoparticles (NPs) composed of a plasmonic Au core and a bimetallic, alloyed plasmonic-catalytic AuPd shell (Au@AuPd). These NPs possess low Pd content that provides the ideal conditions to marry the advantages of strong plasmonic properties, attributed to the LSPR excitation from the Au cores, with enhanced utilization of catalytic metal (the Pd loading corresponded to 3 or 0.3 atom %). Our spectroscopic and electron microscopy investigations showed that by controlling the Pd content in these samples, similar shell thicknesses for the alloys at the Au surface were detected (4 or 5 atomic layers in Au_99.7_Pd_0.3_ and Au_99_Pd_3_ NPs, respectively). As the Pd content decreases, the Pd distribution within the alloyed shells becomes more dilute. This precise manipulation of the Pd distribution enabled optimization of the catalytic activity and increased reaction selectivity under both dark and visible light irradiation conditions due to plasmonic effects. By employing the production of NH_3_ from NO_2_^−^ (nitrite reduction reaction, NO2RR) as a model transformation, an increase of 10-fold and 11-fold in mass activity was detected in the dark and light illumination conditions, respectively, for Au_99.7_Pd_0.3_ relative to Au_97_Pd_3_ NPs. CO-TPD and DFT results showed that this optimization of the Pd distribution in Au_99.7_Pd_0.3_ enabled stronger interactions with adsorbed NO and decreased energy barriers for the NO2RR. Moreover, the presence of pronounced electron accumulation regions at the Pd sites in Au_99.7_Pd_0.3_ facilitates the effective transfer of LSPR-excited hot electrons to the Pd sites, contributing to the plasmonic enhancements in the NO2RR. The tuning over Pd concentration in the surface AuPd alloys and the plasmonic effect in Au_99.7_Pd_0.3_ also enabled control over reaction selectivity, with an increase in the formation of NH_3_ both in the dark and under light excitation conditions relative to that of Au_97_Pd_3_. From Au_99_Pd_3_ to Au_99.7_Pd_0.3_, the reaction selectivity toward the formation of NH_3_ under light excitation increased from 3.8 to 5.8 mg L^−1^h^−1^. This increase in reaction selectivity can be assigned to the larger extent of hydrogenation enabled by the more dilute Pd distribution in the shells of Au_99.7_Pd_0.3_ while suppressing the pathway that leads to N_2_ (which requires coupling between two adjacent surface N-containing species). We believe that the results presented herein can provide important insights into the rational design of antenna-reactor plasmonic-catalytic NPs with improved activities and selectivity for molecular transformations related to sustainability, which is crucial to achieving the world’s net zero goals.

## Methods

### Materials and Instrumentation

Chloroauric acid trihydrate (HAuCl_4_·3H_2_O, 99.9%, Sigma-Aldrich), potassium hexachloropalladate (IV) (K_2_PtCl_6_, 99.99%, Sigma-Aldrich), trisodium citrate dihydrate (C_6_H_5_Na_3_O_7_·2H_2_O, 99%, Sigma-Aldrich), l-ascorbic acid (C_6_H_8_O_6_, 99%, Sigma-Aldrich), nitric acid (HNO_3_, 65.0−67.0%, Sigma−Aldrich), silica nanopowder (SiO_2_, Sigma-Aldrich), isopropanol (C_3_H_8_O, HPLC grade 99.9%, Sigma−Aldrich), Vulcan XC-72R (FullCellStore), perchloric acid (HClO_4_, ACS reagent, 70%, Sigma−Aldrich), and sodium nitrite (NaNO_3_, trace metals basis 99.999%, Sigma-Aldrich) were used as received. Deionized water (18.2 MΩ•cm Milli-Q system) was used for synthesis and support electrolyte preparation.

Transmission electron microscopy (TEM) images were acquired on a Jeol JEM-1400 TEM. TEM samples were prepared by dispersing the nanoparticle suspension in deionized (DI) water with an ultrasonic bath and drop casting onto carbon-coated copper grids. The histogram of the particle size distribution was determined by individually measuring the diameters of 250 nanoparticles. The suspension containing the NPs was drop cast onto an oxidized Si wafer and dried under ambient conditions before imaging. UV−vis spectra were acquired directly from the NP aqueous suspensions using a Shimadzu UV-2600 spectrometer from 800 to 200 nm with a step size of 1 nm.

Powder X-ray diffraction (PXRD) data of silica-supported NPs was collected on a Bruker D8 Advance in Bragg−Brentano geometry using Cu Kα radiation (λ = 1.5406 Å) with a Ni filter. Diffraction data were collected over a range of 10−70° 2θ (step width 0.02° 2θ, count time 1 s/step). The diffraction patterns have been indexed by comparison with the Joint Committee on Powder Diffraction Standard (JCPDS) files. The elemental composition analysis was performed by Microwave Plasma Atomic Emission Spectroscopy (MP-AES) using Agilent Technologies 4100 MP-AES. Three independent measurements were performed for each sample.

X-ray photoelectron spectroscopy (XPS) was performed using a PREVAC spectrometer with a monochromatized Al Kα anode (1486.7 eV) under an ultrahigh vacuum (10^−10^ mbar). The samples were prepared by drop casting the corresponding suspension containing the NPs onto Si support, followed by drying under ambient conditions. The survey spectra were measured with 200 eV pass energy, and high-resolution spectra were measured with 100 eV pass energy. Casa XPS software was used for data interpretation, and the Shirley background method was employed in the deconvolution process. Some X-ray photoelectron spectra were recorded with a lab-based spectrometer (SPECS GmbH, Berlin) using a monochromated Al Kα source (*hv* = 1486.6 eV) operated at 50 W as the excitation source. In the spectrometer, the X-rays are focused with a μ-FOCUS 600 monochromator onto a 300 μm^2^ spot on the sample, and the data are recorded with a PHOIBOS 150 NAP 1D-DLD analyzer in fixed analyzer transmission (FAT) mode. The pass energy was set to 40 eV for survey scans and 20 eV for high-resolution regions. Charge compensation was required for data collection. Recorded spectra were additionally calibrated against the C 1s internal reference. Data interpretation was performed via Casa XPS. A Shirley or two-point linear background was used depending on the spectrum shape.

Temperature-programmed desorption measurements were performed with a Micromeritics 3Flex 3500 instrument. Before analysis, 50−150 mg of the sample was packed into a quartz U-tube reactor and outgassed in a flow of helium (He) at 100 °C for 30 min. Samples were hydrogenated in a flow of H_2_ (100 °C for 60 min) to achieve a comparable state of reduction prior to measurement. After hydrogenation, the temperature was set to 35 °C under He flow (100 mL/min). At 35 °C, the flow rate was increased to 200 mL/min, and CO adsorption was started with loop injection. The sample CO saturation was monitored with a thermal conductivity detector (TCD), where at least three injections were performed to achieve full saturation. After CO adsorption, the sample was kept in a He flow (200 mL/min) for 30 min before starting temperature-programmed desorption (35 to 400 °C with a ramp rate of 10 °C/min). Desorbed CO was measured with a mass spectrometer (Balzers Omnistar GSD 300 O3) monitoring CO at 28 *m*/*z*.

The high-angle annular dark field (HAADF) scanning transmission electron microscopy (STEM) imaging was performed using a probe-corrected FEI Titan G2 80−200 S/TEM instrument equipped with the Super-X energy-dispersive X-ray fluorescence (EDX) detector and a Gatan Quantum ER imaging filter (GIF) for electron energy loss spectroscopy. The Titan STEM was operated at an accelerating voltage of 200 kV. The HAADF STEM images were acquired using a probe current of 300 pA, convergence semiangle of 21.5 mrad, and a HAADF inner collection angle of 43 mrad. The images were collected with a dwell time of 10 μs, resulting in a total frame time per image of ∼12.6 s. The STEM-EDX spectrum images were collected with a probe current of 300 pA using all 4 EDX detectors for a total acquisition time of ∼10 min. Analysis of the STEM-EDX data was carried out using in-house built Python scripts with packages including Hyperspy v1.6.31.^56^

### Model Fitting for EDX Quantification

Quantification of EDX data was initially attempted with Gatan Digital Micrograph and Bruker ESPRIT software, but neither achieved consistently reliable results, likely due to the exceptionally low alloy content and overlapping spectral features. These traditional EDX analysis approaches only consider the intensity of an individual X-ray peak in the EDX spectra to quantify the elemental compositions (e.g., Au Kα). However, many elements have several peaks visible in the X-ray spectrum, the consideration of which has the potential to reduce experimental error since all peaks should reflect the concentration of the appropriate element. Quantification based on the intensity of all X-ray peaks improves the confidence in the intensity measurement of a single peak. Including all such peaks maximizes the information content, which is particularly important for noisy data sets or for which the content of a particular element is low. To combine the information on different EDX peaks for the same element, it is necessary to fit a model to the sum of the spectrum image (Figure S5). This was achieved using Python-based fitting, which involves applying a model consisting of a polynomial background function and a series of Gaussian peaks to the spectrum. The model was then used to extract the quantification.

EDX error minimization can be accomplished by reducing the width of the integration windows to isolate the peaks as much as possible. In addition, the background subtraction window was moved outside of the characteristic peak energy range into flat areas on either side of the peaks. The approximation of mean shell thickness was calculated using the overall atomic % of Au and Pd, considering that all of the Pd detected was on the surface of the particle in a homogeneous shell layer with a composition of 100% Pd. The calculated shell thicknesses for Au_97_Pd_3_ and Au_99.7_Pd_0.3_ NPs were 280 pm (0.28 nm) and 120 pm (0.12 nm), respectively (Pd atomic radius = 0.137 nm). Alternatively, we can consider a model where the Pd content is uniformly distributed within the measured radially averaged shell thickness, *r*_2_, for a particle with radius, *r*, and a 100% Au core (see Figure S8 for the model). The particles in Figure 3B,E (main text) have *r*_2_ = 1.4 and 1.2 nm and *r* = 10 and 11 nm for the Au_99.7_Pd_0.3_ and Au_97_Pd_3_, respectively, so this perfect two-phase core−shell model gives the shell a composition of 25.3 atom % Pd and 10.2 atom % Pd for Au_97_Pd_3_ and Au_99.7_Pd_0.3_, respectively. However, we note that the distribution of Pd visible in Figures 3 and S6 is not perfectly homogeneous in the shell region, and the morphology is therefore likely better represented by a core−shell structure with the surface shell having a variable Pd enrichment.

### Computational Details

DFT calculations were carried out by the DMol^3^ module of Materials Studio.^57^ The generalized gradient approximation of Perdew−Burke−Ernzerhof exchange–correlation functionals was used to calculate the exchange and correlation energy in this work.^58^ Core treatment was adopted as All Electron to conduct the metal relativistic effect, and the double numerical plus polarization function basis set was used. A smearing of 0.01 Ha (1 Ha = 27.21 eV) to the orbital occupation and 1 × 10^−5^ Ha convergence criterion for self-consistent-field (SCF) calculations were applied. The van der Waals (vdW) interactions were taken into consideration by the Grimme scheme (DFT-D3).^59^ The geometry optimization convergence tolerance for energy change, maximum force, and maximum displacement were 1 × 10^−5^ Ha, 0.004 Ha/Å, and 0.005 Å, respectively. The vacuum spacing in the direction along the *Z* axis, with respect to the surface, was 20 Å between neighboring slab images, which is sufficient to eliminate the interactions between the slabs. During the geometry optimizations, only the top AuPd alloy layer was relaxed and the bottom Au layers were fixed at the bulk positions.

The free energy (Δ*G*) calculations for each elementary step were based on the standard hydrogen electrode model,^60^ and the change in reaction free energy can be obtained with the equation below:
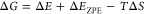
1where Δ*E* is the total energy difference before and after intermediate adsorbed, and Δ*E*_ZPE_ and Δ*S* are the differences of zero-point energy and entropy, respectively.

For the nitrite reduction reaction, the chemical reaction considered can be summarized with the reaction equations below:
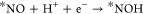
2
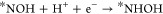
3
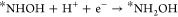
4
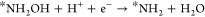
5
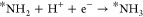
6
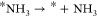
7where * represents the active site. The zero-point energy and entropy of free molecules and adsorbents were obtained from vibrational frequency calculations.

### Synthesis of Au and AuPd NPs

For the synthesis of Au NPs, 100 mg of trisodium citrate (0.34 mmol) was dissolved in 148 mL of deionized water in a round-bottom flask under boiling conditions (110 °C).^61^ Then, 2 mL of HauCl_4_·3H_2_O solution (0.025 mmol) was added to this mixture, which was kept under magnetic stirring for over 30 min, enabling the formation of a red suspension containing Au NPs. AuPd NPs were synthesized by transferring 75 mL of the aqueous Au NP suspension obtained in the previous step to a round-bottom flask, followed by stirring at 70 °C for 20 min using a silicon bath. Then, 35.2 mg of l-ascorbic acid (0.2 mmol) was added to this suspension, followed by stirring for another 30 min. Then, 215 μL (0.658 μmol) or 21.5 μL (0.066 μmol) of K_2_PdCl_4_ solution (1 mg/mL) was added to the reaction mixture to form Au_97_Pd_3_ and Au_99.7_Pd_0.3_ NPs, respectively. Following the addition of the Pd precursor, the reaction mixture was kept at 70 °C with stirring for another 30 min. The NPs were then washed with water by successive rounds of centrifugation and removal of the supernatant. We also prepared AuPd NPs supported on SiO_2_ (3 wt % in terms of metal) to perform XRD and CO-TPD characterization. In this case, the pH of the AuPd NP suspension obtained in the previous step was adjusted to 3 by the addition of 20 μL of concentrated HNO_3(aq)_. This was followed by the addition of 100 mg of nanosilica powder and stirring for 12 h at 70 °C to produce AuPd/SiO_2_ samples. The AuPd/SiO_2_ NPs were isolated by centrifugation for 20 min at 7500 rpm and washed twice with water by successive rounds of centrifugation and removal of the supernatant, and the mixture was dried at 70 °C.

### Electrocatalytic Studies: Nitrite Reduction Reaction (NO_2_RR)

Electrochemical experiments were performed in a three-electrode glass cell, with a glassy carbon rod (GCE) used as a working electrode (6 mm diameter, geometric area of 0.2827 cm^2^) and a high area graphite rod used as a counter electrode. All of the potentials were measured and displayed based on the reversible hydrogen electrode (RHE) prepared with the same solution of the supporting electrolyte (0.1 M HClO_4_). The GCE electrode was purified by polishing with alumina slurry and sonicating in ultrapure water and acetone (5 min each). To avoid residual contamination from the synthesis, the NPs were cleaned twice by washing them with water to remove synthesis reactants. After that, the clean GCE was modified by drop casting 30 μL of catalyst ink (AuPd nanoparticles and carbon black Vulcan XC−72R dispersed in H_2_O: IPA solution), resulting in a uniform film with a Au loading of 200 μg/cm^2^ and Vulcan carbon loading of 100 μg/cm^2^.

The electrochemical measurements were carried out at room temperature (25 °C), using an Autolab PGSTAT 128 N equipped with a Scan 250 modulus as a potentiostat. Before the experiments, the solution was purged with Argon 2.2, which was kept in the cell headspace during the data collection. The plasmonic excitation was performed by irradiating the electrochemical cell with one Kessil PR 160L LED with 525 nm as the light wavelength (total irradiance of 59.50 mW cm^−2^). For electrocatalytic studies, cyclic voltammograms were normalized by the palladium mass present in each catalyst, according to the results of atomic emission spectroscopy experiments (MP-AES). The data normalized by the geometric area are also provided.

For the NO_2_RR studies, the supporting electrolyte was prepared by diluting HClO_4_ directly into 18.2 MΩ cm of water. Sodium nitrite was then added to the electrolyte from a 1 M stock solution to achieve the target concentration as described in the text. Cyclic voltammetry (CV) measurements were performed at a scan rate of 10 mV s^−1^, and chronoamperometric analysis (CA) measurements were recorded at 0.050 V (vs RHE) under chopped illumination, both in Ar-saturated 0.1 M HClO_4_ solution.

The indophenol method was used to determine the NH_3_ concentration produced by the nitrite conversion.^33^ The CA measurements were performed at 0.05 V for 1 h using an electrochemical cell with a controllable warm water bath at 25 °C through a Julabo F12−MA refrigerated circulator to avoid the temperature variation. Briefly, considering that the indophenol reaction is pH-dependent, sodium hydroxide solution was added to the electrolyte to achieve an alkaline pH. Five mL portion of electrolyte was mixed with 600 μL of 2.75 M sodium salicylate and 0.95 mM sodium nitroprusside solution. The resultant solution was kept in the dark for 45 min after the addition of 1 mL of solution containing 306 mM sodium citrate, 418 mM sodium hydroxide, and 1.5 mM sodium hypochlorite (10% active chlorine basis). The indophenol dye formed after the reaction between ammonium ion and salicylate (see the mechanism proposed by Krom^3^) can be determined by the 650 nm band in the UV−Vis spectra. The absorbance at 650 nm was plotted against a calibration curve to calculate the NH_3_ concentration.

## Supporting Information Available

Additional characterization (MP-AES, electron microscopy data and modeling, histograms of size distribution, XPS, LSVs); catalytic data (LSVs, UV−vis); and computational modeling (PDF)
