# Supplementary material for: Au@AuPd Core-Alloyed Shell Nanoparticles for Enhanced Electrocatalytic Activity and Selectivity under Visible Light Excitation
Source: ACS Nano. 2024 Aug 20;18(35):24391–403. doi: 10.1021/acsnano.4c07076 (PMC11386439; doi:10.1021/acsnano.4c07076)
Supplement: Supplementary file 1 — nn4c07076_si_001.pdf [file nn4c07076_si_001.pdf]

Supporting Information for

Au@AuPd core-alloyed shell nanoparticles for enhanced electrocatalytic activity and selectivity under visible light excitation

Kaline N. da Silva,[1] Shwetha Shetty,[1] Sam Sullivan–Allsop,[2] Rongsheng Cai,[2] Shiqi Wang,[1] Jhon Quiroz,[1] Mykhailo Chundak,[1] Hugo L. S. dos Santos,[1] IbrahiM Abdelsalam,[1] Freddy E. Oropeza,[3] Víctor A. de la Peña O'Shea,[3] Niko Heikkinen,[4] Elton Sitta,[5] Tiago V. Alves,[6] Mikko Ritala,[1] Wenyi Huo,[7,8*] Thomas J. A. Slater,[9] Sarah J. Haigh,[2*] and Pedro H.C. Camargo[1,*]

[1]*Department of Chemistry, University of Helsinki, A.I. Virtasen aukio 1, PO Box 55, FIN-0014 Helsinki, Finland.*

[2] *Department of Materials, University of Manchester, Manchester M13 9PL, United Kingdom.*

[3] *Photoactivated Processes Unit, IMDEA Energy Institute, Avda. Ramón de la Sagra 3, 28935 Mostoles, Madrid, Spain.*

[4] *VTT Technical Research Centre of Finland, P O Box 1000, FIN-02044 Espoo, Finland*

[5] *Department of Chemistry, Federal University of Sao Carlos, Rod. Washington Luis, km 235, Sao Carlos, 13565–905, Brazil.*

[6] *Departamento de Físico-Química, Instituto de Química, Universidade Federal da Bahia, Rua Barão de Jeremoabo, 14740170-115 Salvador-BA, Brazil*

[7] *College of Mechanical and Electrical Engineering, Nanjing Forestry University. Nanjing, 210037, P. R. China.*

[8] *NOMATEN Centre of Excellence, National Centre for Nuclear Research. Otwock, 05-400, Poland*

[9] *Cardiff Catalysis Institute, School of Chemistry, Cardiff University, Cardiff, CF10 3AT, United Kingdom.*

** Corresponding authors.* Email: pedro.camargo@helsinki.fi, sarah.haigh@manchester.ac.uk, wyhuo@njfu.edu.cn.

Table S1. Atomic and weight % composition of obtained from MP-AES.

Catalyst	Au at.%	Pd at. %	Pd. wt.%
$Au_{97}Pd_3/SiO_2$	97.0	3	0.05
$Au_{99.7}Pd_{0.3}/SiO_2$	99.7	0.3	0.005

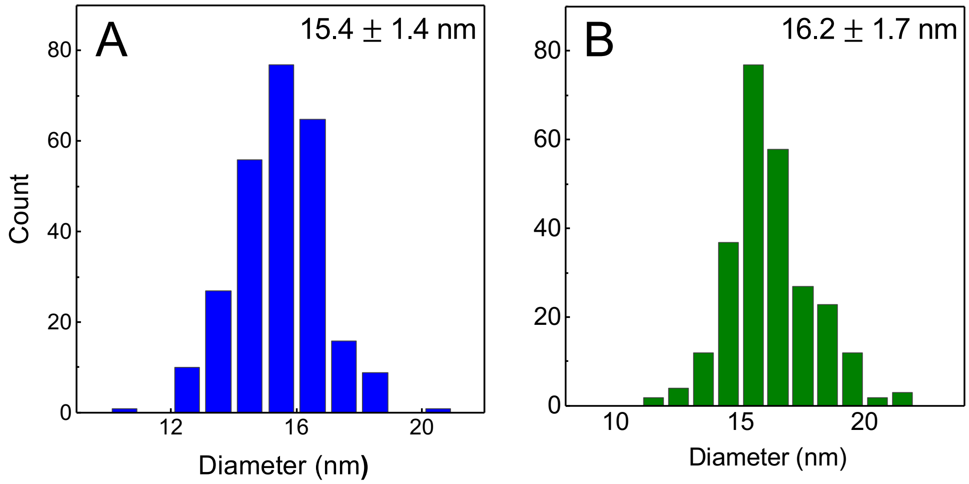

Figure S1. Histograms of size distribution for (A) $Au_{99.7}Pd_{0.3}$ and (B) $Au_{97}Pd_3$ NPs, each measured from TEM images of 250 particles

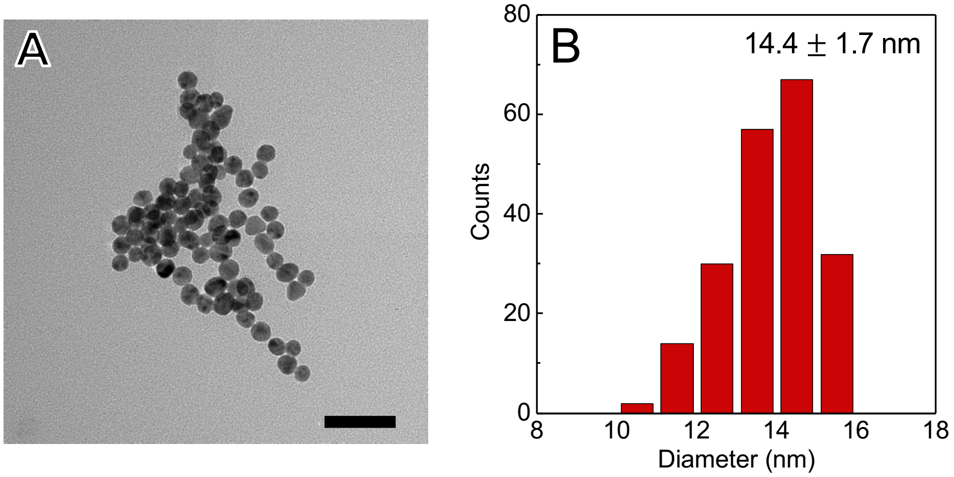

Figure S2. TEM image and histogram of size distribution for the Au NPs employed as seeds for the synthesis of $Au_{99.7}Pd_{0.3}$ and $Au_{97}Pd_3$ NPs. Histogram measured from TEM images of 250 particles. Scale bar corresponds to 50 nm.

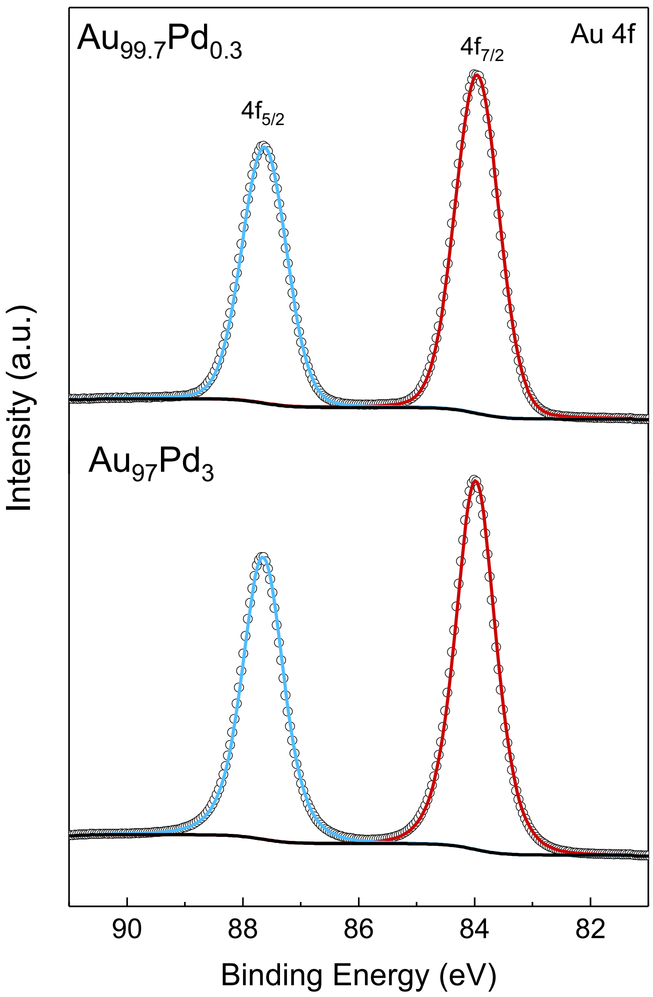

Figure S3. High–resolution XPS spectra of the Au 4f core levels for $Au_{99.7}Pd_{0.3}$ and $Au_{97}Pd_3$ NPs (top and bottom traces, respectively).

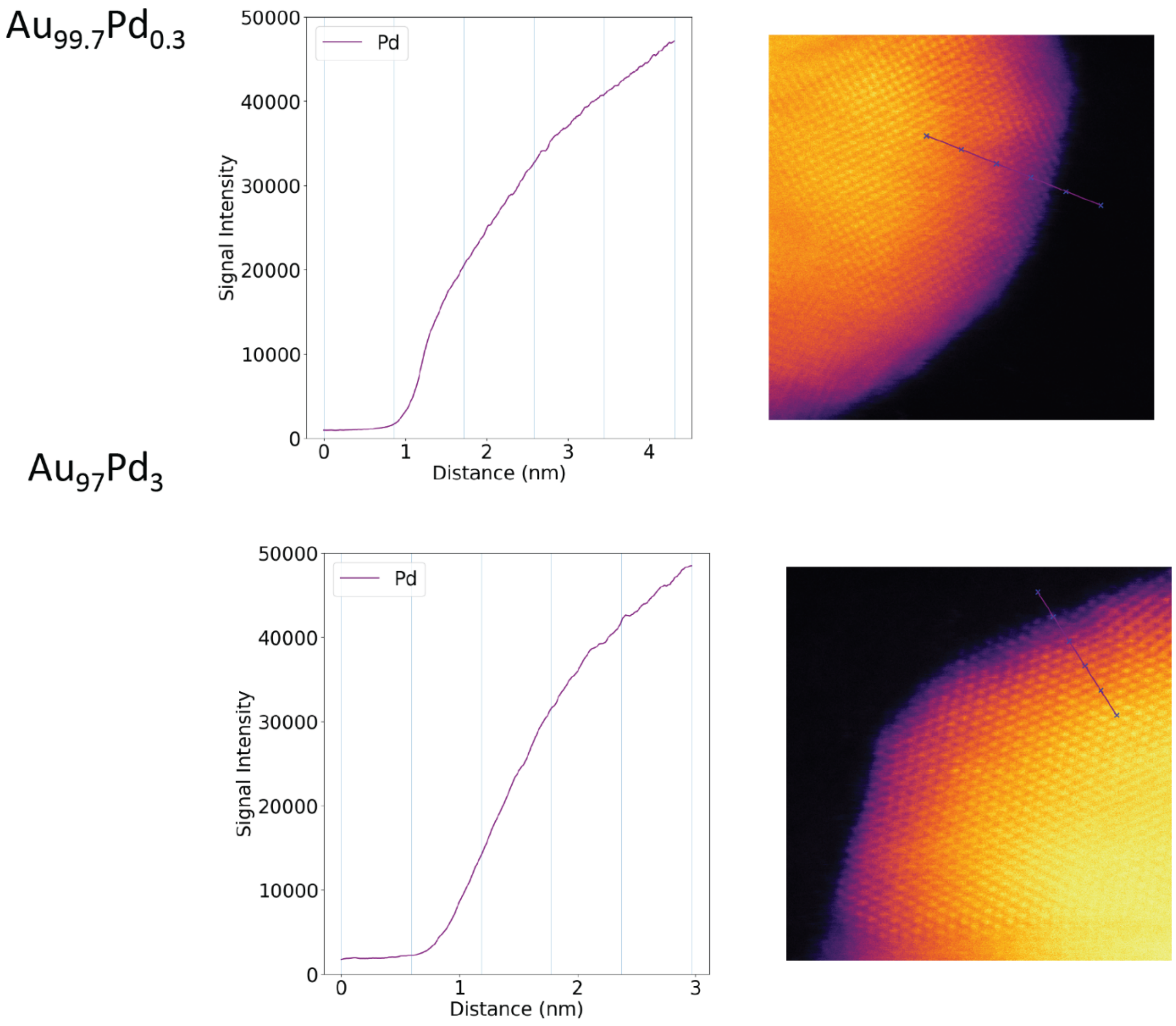

Figure S4. STEM-HAADF linescans (left panels) from the STEM-HAADF images (right panels) for the locations indicated by the black lines shown in the STEM-HAADF images for (top panels) $Au_{99.7}Pd_{0.3}$ and (bottom panels) $Au_{97}Pd_3$ NPs. The linescans are averaged over a width of 40 pixels.

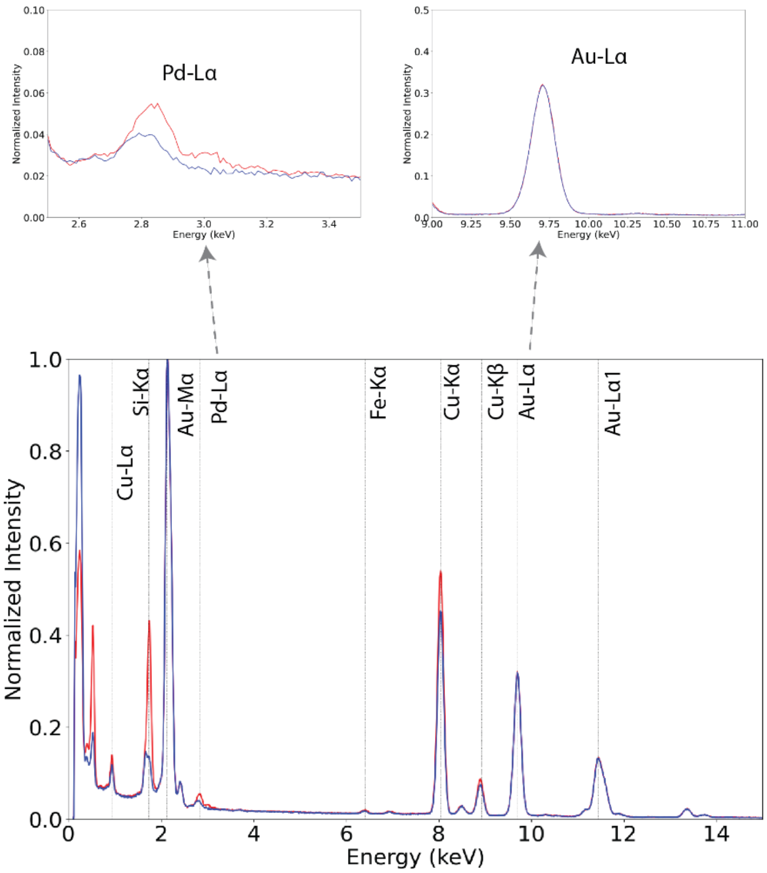

Figure S5. Summed EDX spectra for the $Au_{97}Pd_3$ and the $Au_{99.7}Pd_{0.3}$ NPs (bottom panel). Extracted regions around the Pd Lα and Au Lα peaks (top panels) respectively are displayed for clarity, where the expected peak positions are indicated by the grey lines. The spectra are shown normalized to the Au Lα peak. The Cu–Kα peak is also present and is an artefact from the presence of the Cu support grid. A small peak from Fe is also an artefact of electron/X-ray scattering from the TEM polepiece.

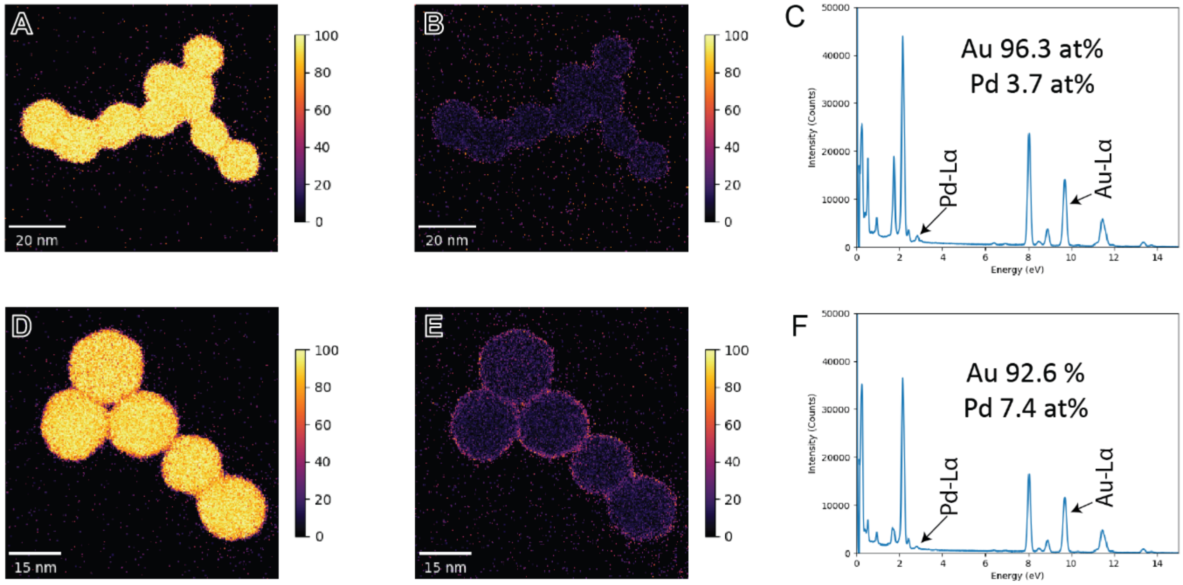

Figure S6. STEM-EDX elemental mapping for (A and D) Au and (B and E) Pd, and the respective EDX spectra for (C) $Au_{99.7}Pd_{0.3}$ and (F) $Au_{97}Pd_{3}$ NPs. Note that the Pd counts seen in the centre of the particle are not evidence of internal Pd but likely result from the transmission nature of the technique, with the centre of the particle sampling the top and bottom surfaces.

$Au_{99.7}Pd_{0.3}$

Sample	Au %	Pd %
09	96.270	3.729
10	95.710	4.290
11	95.945	4.055
12	95.991	4.009
13	96.598	3.402
14	97.013	2.987
Average	96.255	3.745
Standard Deviation	0.44	0.44

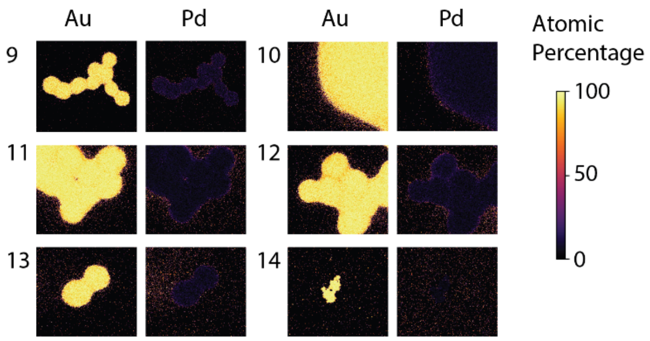

$Au_{97}Pd_{3}$

Sample	Au %	Pd %
01	92.62	7.38
02	93.24	6.76
03	92.87	7.13
04	91.76	8.24
05	93.11	6.89
06	92.58	7.42
07	92.90	7.10
08	93.18	6.82
Average	92.66	7.22
Standard Deviation	0.45	0.45

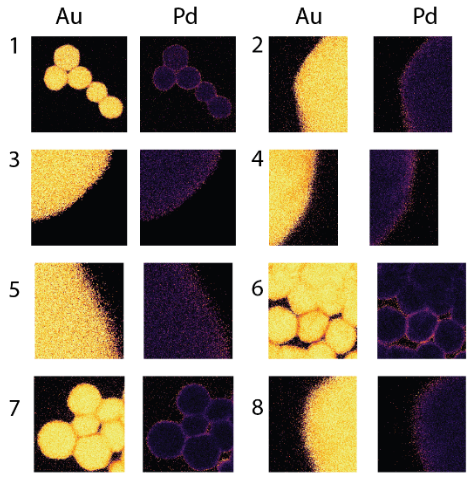

Figure S7. Comparison of semi–quantitative compositions for the normalized summed EDX spectra for $Au_{99.7}Pd_{0.3}$ and $Au_{97}Pd_{3}$ NPs.

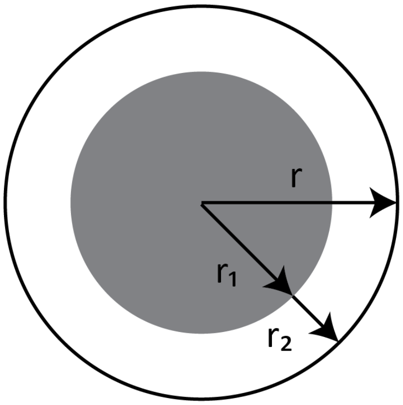

Calculation used:

$$Shell\ \% = \frac{Total\ metal - Metal\ in\ the\ Core}{Shell\ Volume}$$

$$Shell\ \% = \frac{Overall\ \%\ \times r^3}{r^3 - (r - r_2)^3}$$

Figure S8: Schematic representation and equation used for shell composition. A model for the NP shell composition with all the Pd contained within the measured shell thickness (r_2 is 1.4 and 1.2 nm for the $Au_{99.7}Pd_{0.3}$ and $Au_{97}Pd_3$ respectively and r is 7.6 and 8.1 nm, espectively).

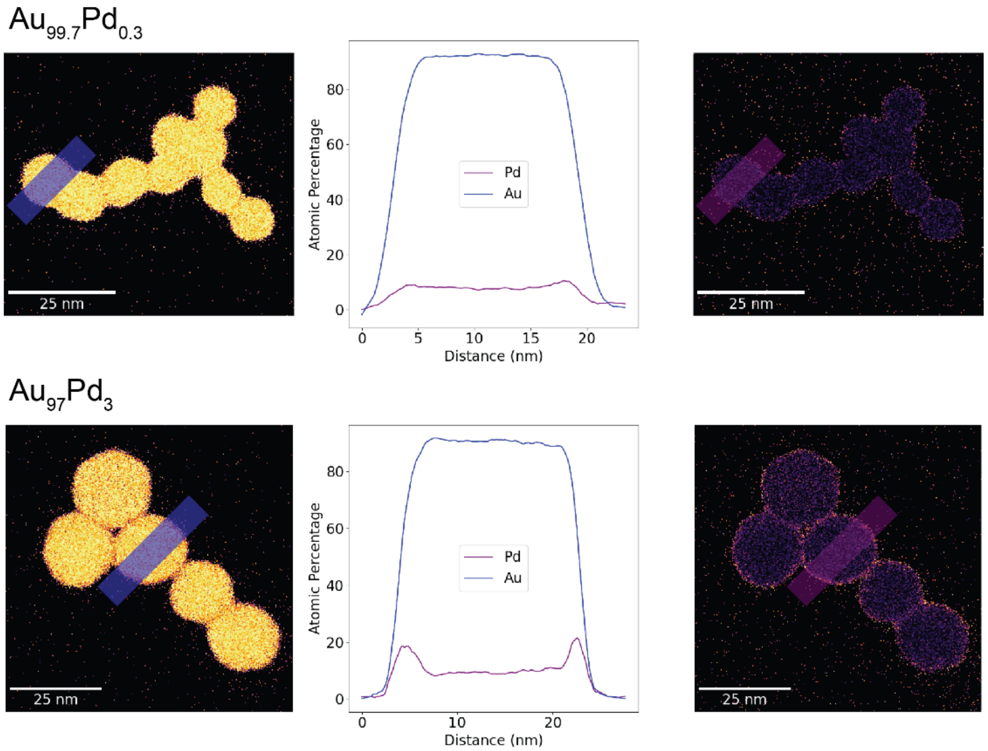

Figure S9. STEM-EDX elemental mapping for (left panels) Au and (right panels) Pd, and (middle panel) the respective EDX line scans for (top) $Au_{99.7}Pd_{0.3}$ and (bottom) $Au_{97}Pd_3$ NPs. The line–scan signal averaged over the width of the line (~2 nm). Note that the Pd counts seen in the centre of the particle are not evidence of internal Pd but likely result from the transmission nature of the technique with the centre of the particle sampling the top and bottom surfaces.

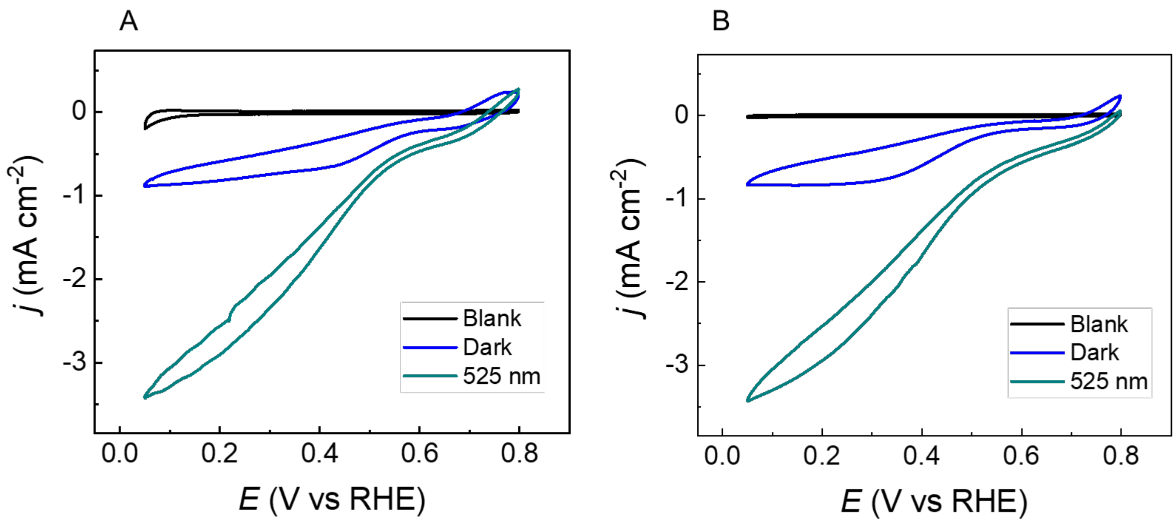

Figure S10. Cyclic voltammograms for (A) $Au_{97}Pd_3$ and (B) $Au_{99.7}Pd_{0.3}$ NPs in 0.1 mol L^{-1} $HClO_4$ (black) and 0.010 mol L^{-1} $NaNO_2$ (blue) registered under blank (electrode only), dark, and 525 nm irradiation conditions. All the voltammograms were recorded at 0.010 V s^{-1}.

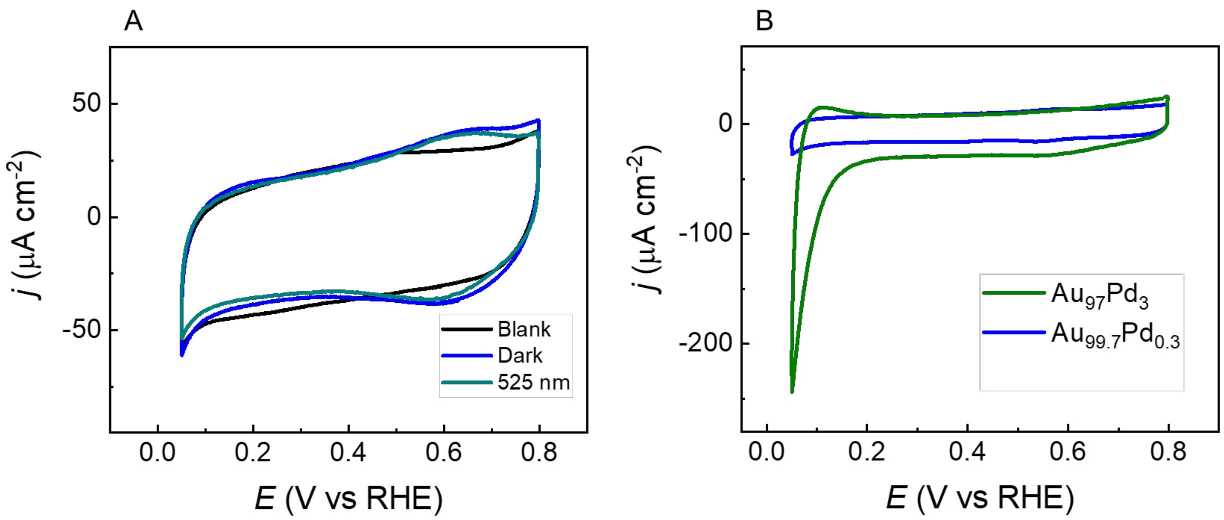

Figure S11. (A) Cyclic voltammograms on Au NPs registered in 0.1 mol L^{-1} $HClO_4$ (black trace), in 0.1 mol L^{-1} $HClO_4$ and 0.050 mol L^{-1} $NaNO_2$ under dark conditions (blue trace), and in 0.1 mol L^{-1} $HClO_4$ and 0.050 mol L^{-1} $NaNO_2$ under light irradiation conditions (green trace). (B) Cyclic voltammograms registered for $Au_{97}Pd_3$ (green trace) and $Au_{99.7}Pd_{0.3}$ (blue trace) in 0.1 mol L^{-1} $HClO_4$ at 0.010 V s^{-1}. All the voltammograms were recorded at 0.010 V s^{-1}.

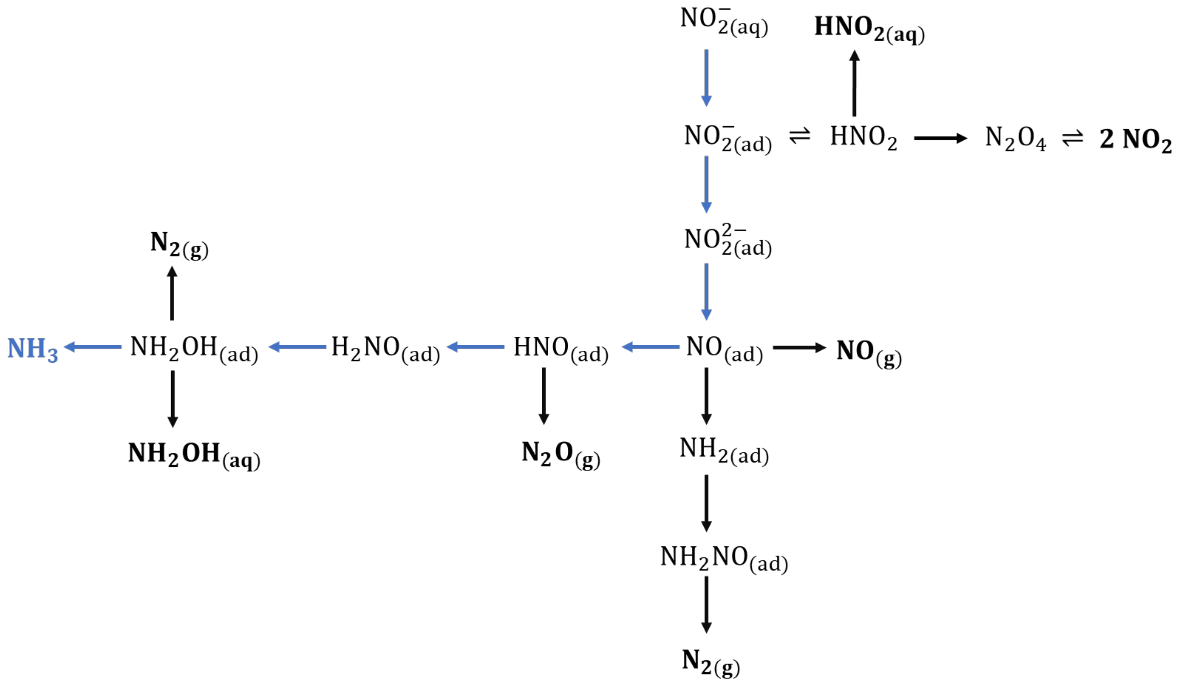

Figure S12: Scheme for the NO_2^- reduction reaction mechanism using noble metals as catalysts. Blue arrows indicate ammonia formation pathway. Possible products are indicated in bold. The conversion of NO_2^- into NH_3 is a six–electron process with a standard potential of +0.792 V (vs. SHE). Its activity increases with the overpotential, with higher current densities observed near 0.05 V where NO_{ad} reduction occurs. Notably, NO_{ad} species play an important role in NO_2^- reduction as their binding energy is stronger than that of NO_2^- on Pt surfaces. Furthermore, NO adlayers are directly formed on transition–metals from acidic nitrite solution, suggesting that NO is likely the electroactive species to be reduced in our experimental conditions.

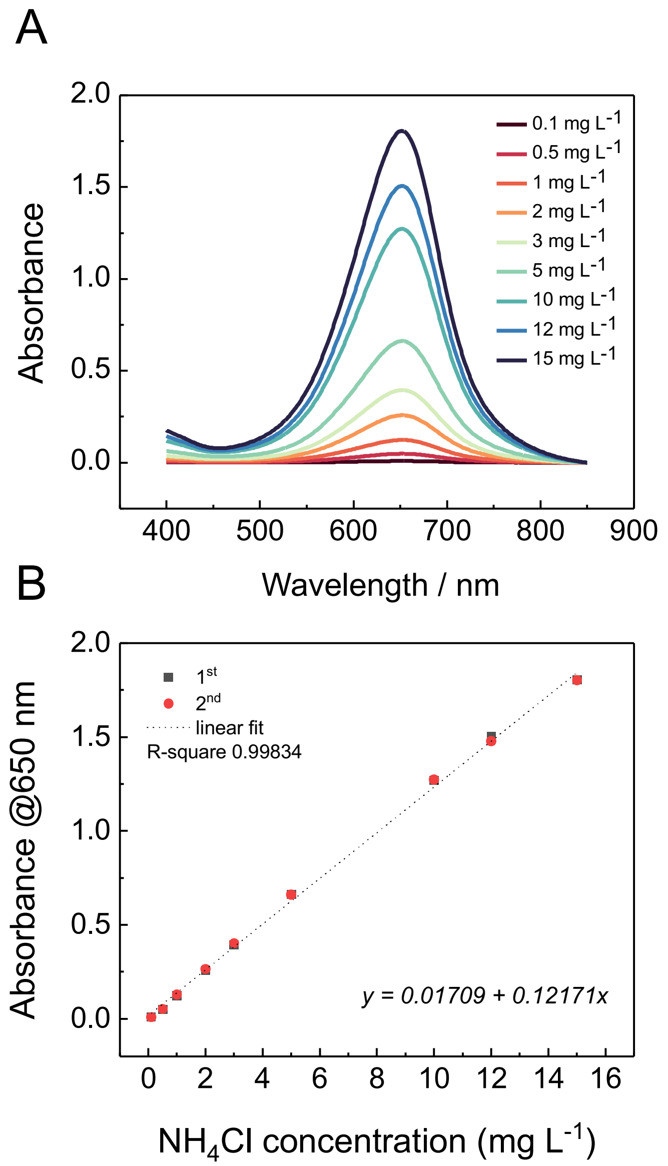

Figure S13. (A) UV-VIS spectra and (B) calibration curves showing the absorbance at 650 nm plotted as a function of $[NH_4Cl]_{aq}$ in the sample according to the indophenol method for the determination of NH_3. Gray squares and red dots correspond to the experimental points, while the dotted line is the fitted linear regression.

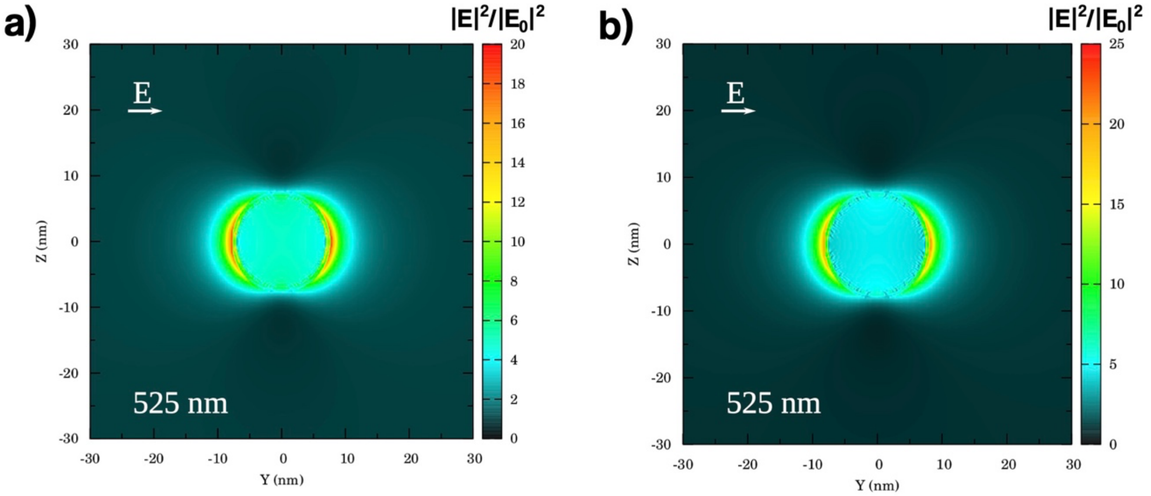

Figure S14. Electric field enhancement contours $|E|^2/|E_0|^2$ calculated by the DDA method for a single (a) $Au_{99.7}Pd_{0.3}$ and (b) $Au_{97}Pd_3$ NP in water. The sizes and compositions were set according to the experimental data (electron microscopy results).The excitation wavelength used in the near-fields calculations was 525 nm, and the polarization direction for sphere and shell along the y-axis. The dielectric constants for Au and AuPd alloy were obtained from the literature.[1,2] The DDA Convert Tool[3] was used to convert a non-standard geometry to a collection of dipoles used by the DDSCAT 7.3.[4] $|E|^2max/|E_0|^2$ values were 19.1 and 21.0 for $Au_{99.7}Pd_{0.3}$ and $Au_{97}Pd_3$, respectively. We employed a spacing of 0.5 nm for the cubic grid. The near fields were described by a grid of 2.5 x 104 points on the yz plane.

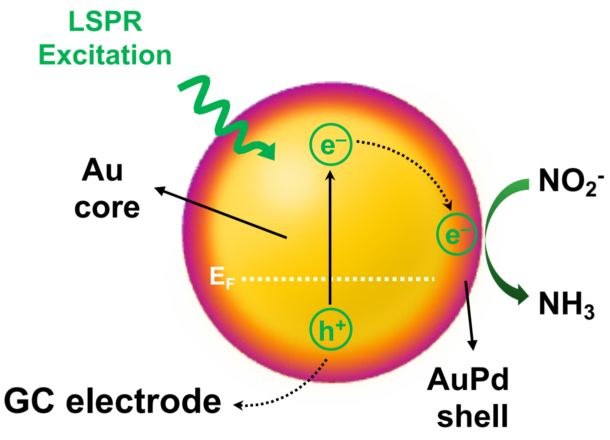

Figure S15. Schematic representation of the LSPR–enhanced NO2RR over AuPd nanoparticles. LSPR excitation leads to the generation of hot carriers, which is also accompanied by an increase in local temperature due to photothermal effects during LSPR relaxation. The hot carriers generated by the Au NPs can transfer to the AuPd shell, leading to an increase in catalytic activity by the activation of surface adsorbates by excited hot electrons. The holes are transported to the counter electrode with the assistance of external voltage.

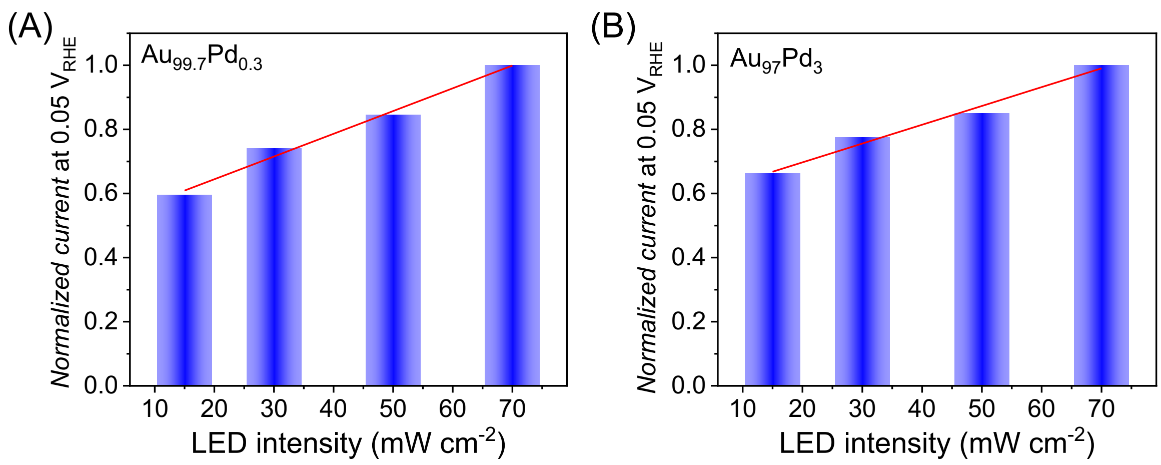

Figure S16. Current densities plotted as a function of light intensity under the same conditions as described in Figure 4 (under 525 nm LED irradiation) for (A) $Au_{99.7}Pd_{0.3}$ and (B) $Au_{97}Pd_3$.

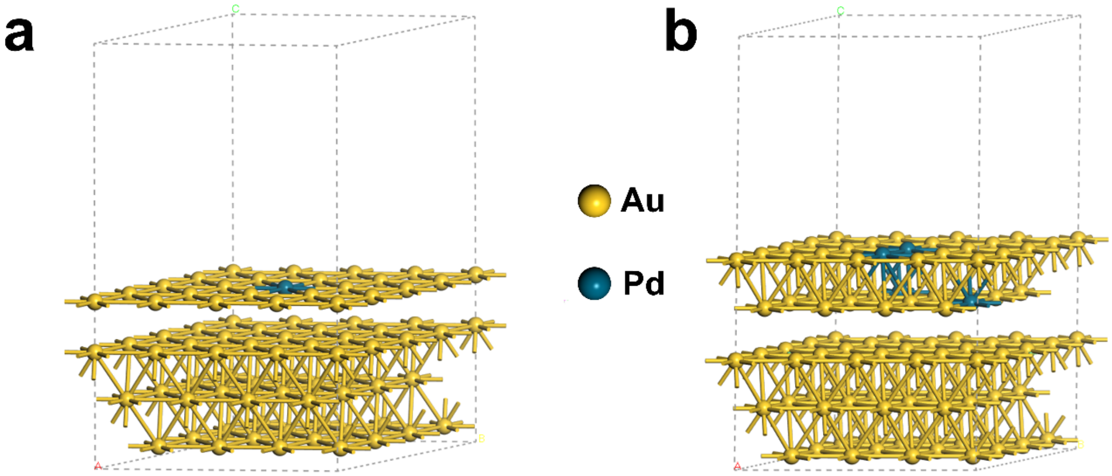

Figure S17. Structural models employed in our calculations for the (a) $Au_{99.7}Pd_{0.3}$ and (b) $Au_{97}Pd_3$ NPs. The AuPd alloy shell in the $Au_{99.7}Pd_{0.3}$ and $Au_{97}Pd_3$ NPs were denoted as shell*1 and shell*2, respectively.

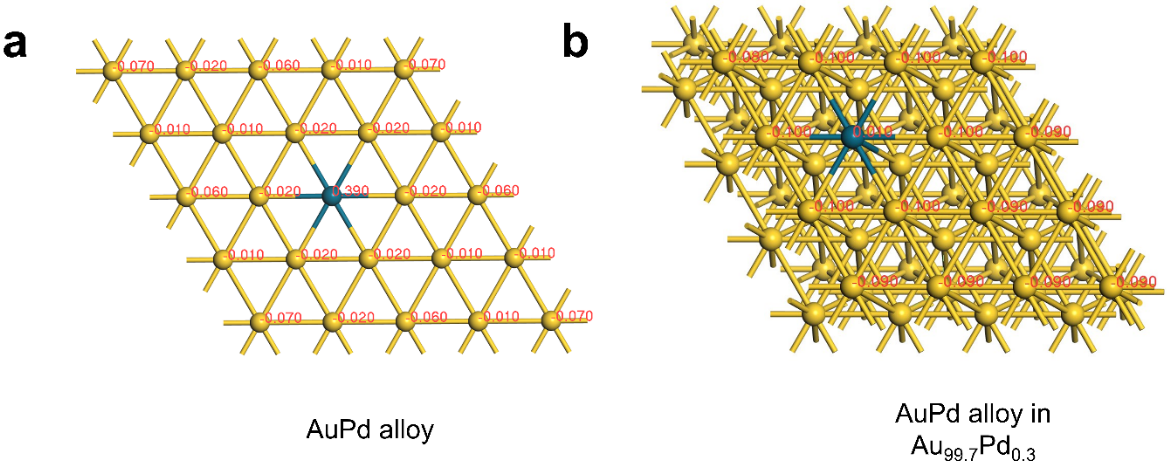

Figure S18. Mulliken charge analysis for (a) an individual Pd atom in AuPd alloy with the expected composition of $Au_{99.7}Pd_{0.3}$ NPs and (b) a AuPd alloy in the $Au_{99.7}Pd_{0.3}$ NPs model (Au core and AuPd alloyed shell two atoms thick).

Table S2. Mulliken charge distributions of Pd and Au atoms in different AuPd alloy shell with/without a core-shell structure, as shown in **Figure S14**.

**AuPd alloy**				
Mulliken Charge	Pd	Au1	Au2	Au3
	0.390	-0.020	-0.020	-0.020
	Au4	Au5	Au6	
	-0.020	-0.020	-0.020	

**AuPt alloy shell in $Au_{99.7}Pd_{0.3}$**				
Mulliken Charge	Pd	Au1	Au2	Au3
	0.010	-0.100	-0.100	-0.100
	Au4	Au5	Au6	
	-0.100	-0.100	-0.100	

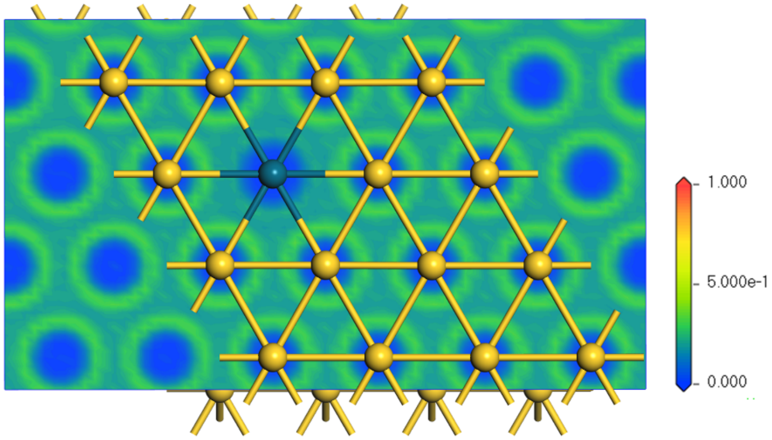

Figure S19. Two-dimensional (2D) ELF evaluation for the AuPd alloy layer in the $Au_{99.7}Pd_{0.3}$ NPs model.

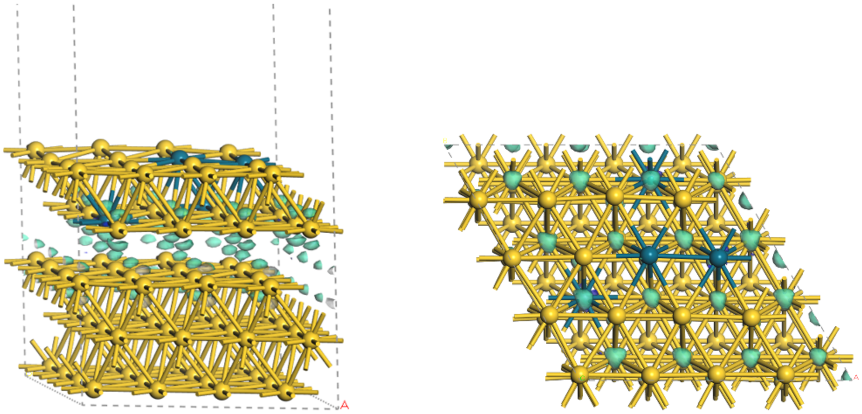

Figure S20. Charge density differences in the constructed $Au_{97}Pd_3$ NPs model (side and top views).

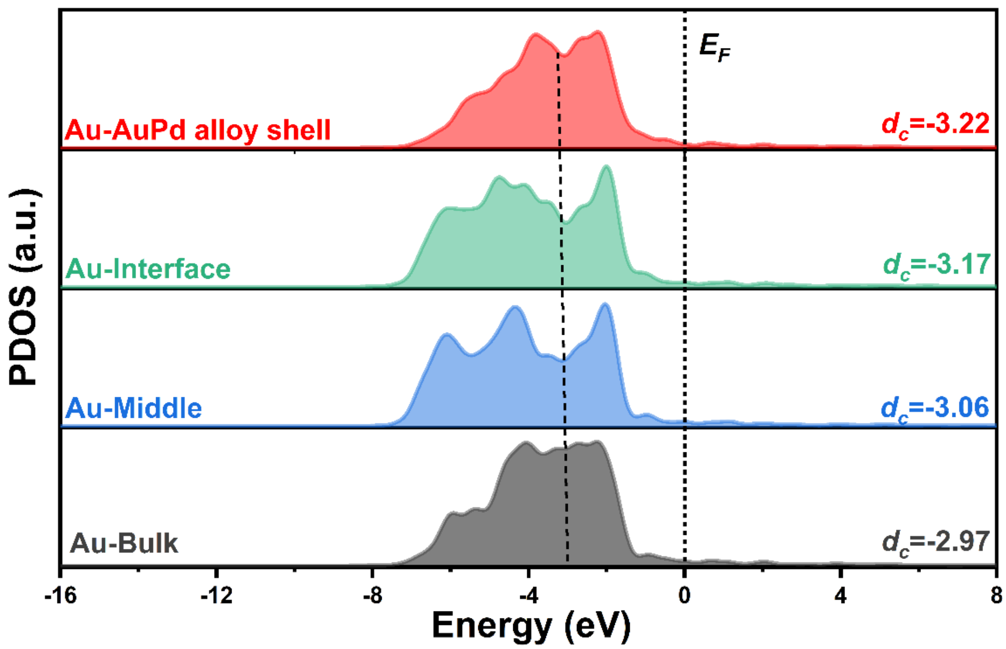

Figure S21. Site-dependent PDOSs of Au-5d in $Au_{99.7}Pd_{0.3}$ NPs model comprising the Au-AuPd interface.

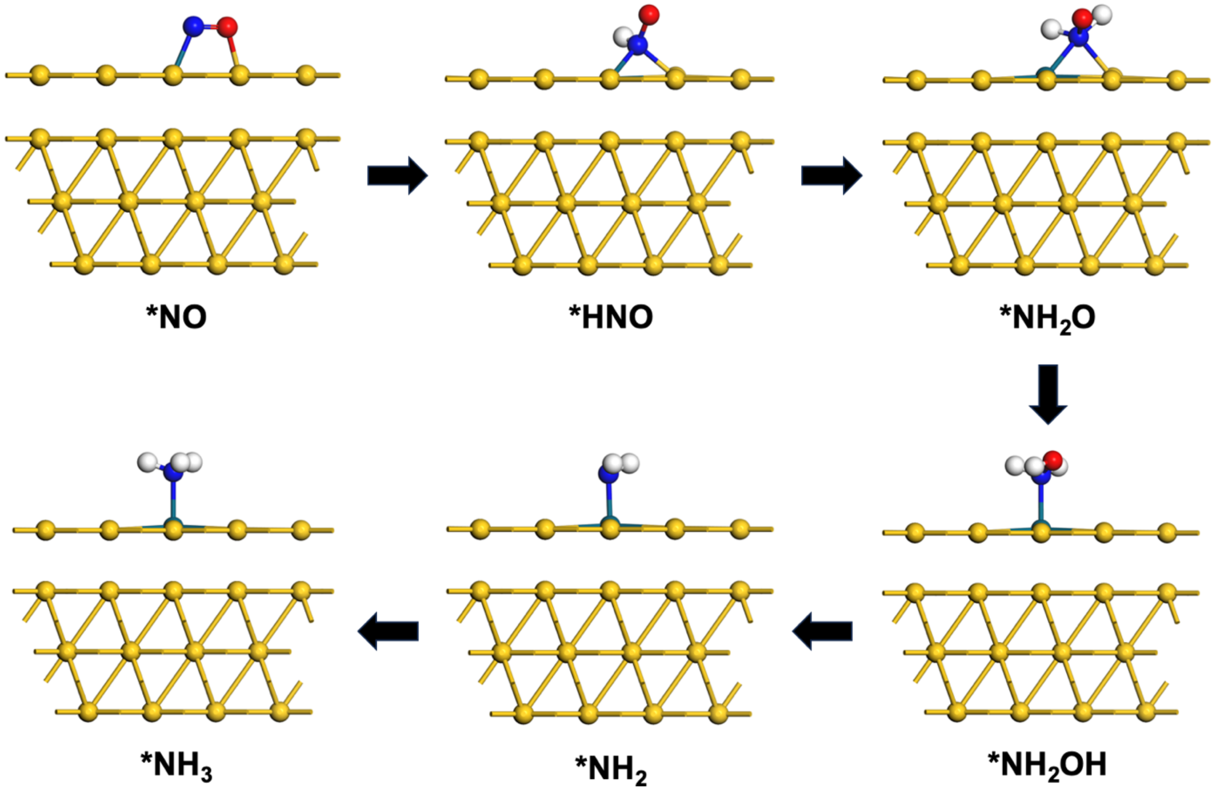

Figure S22. The corresponding configurations for all the intermediates during the NO_2RR on the $Au_{99.7}Pd_{0.3}$ NPs model.

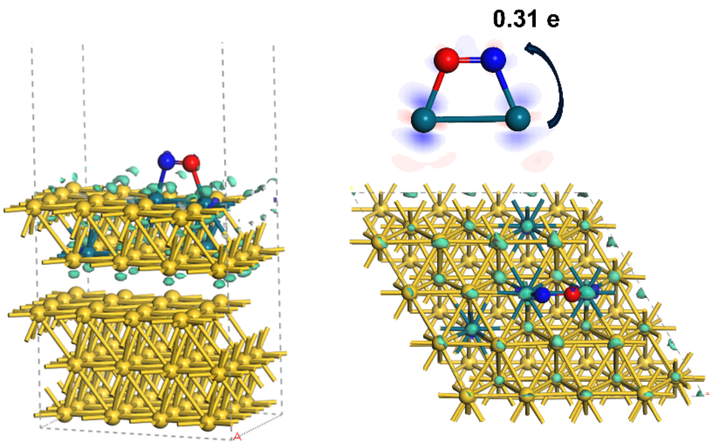

Figure S23. Charge density differences and Mulliken charge analysis results for the constructed $Au_{97}Pd_3$ NPs model containing a NO molecule adsorbed on the surface sites. The blue and bright green contours represent the regions of electron accumulation and depletion, respectively. For 2D maps, the scale from blue to red was -0.4 to 0.4 e.

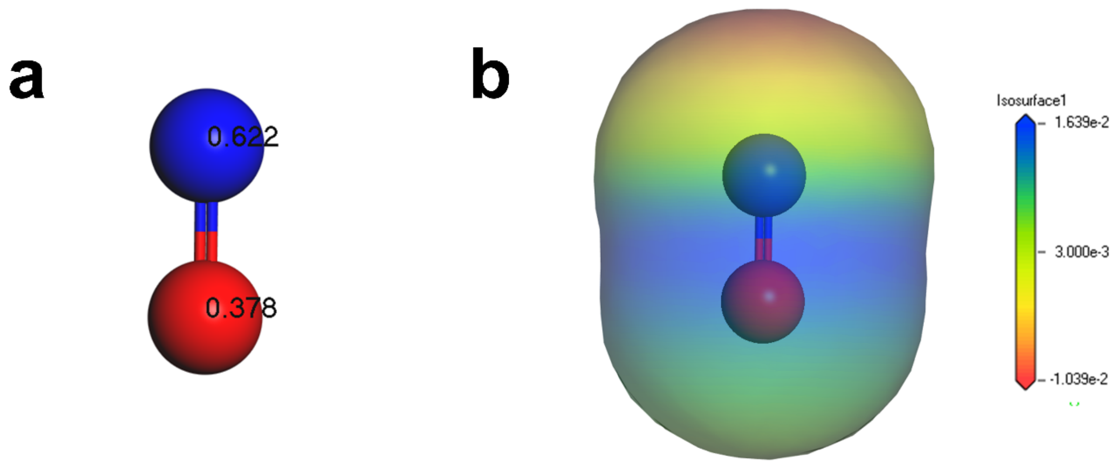

Figure S24. Structural model of the NO molecule with the (a) Fukui charge and (b) electrostatic potential distribution. Due to the large Fukui charge and electrostatic potential of the labeled N atom, we choose this corresponding Pd-N bond (after adsorption) for the further COHP analysis (**Figure S22**).

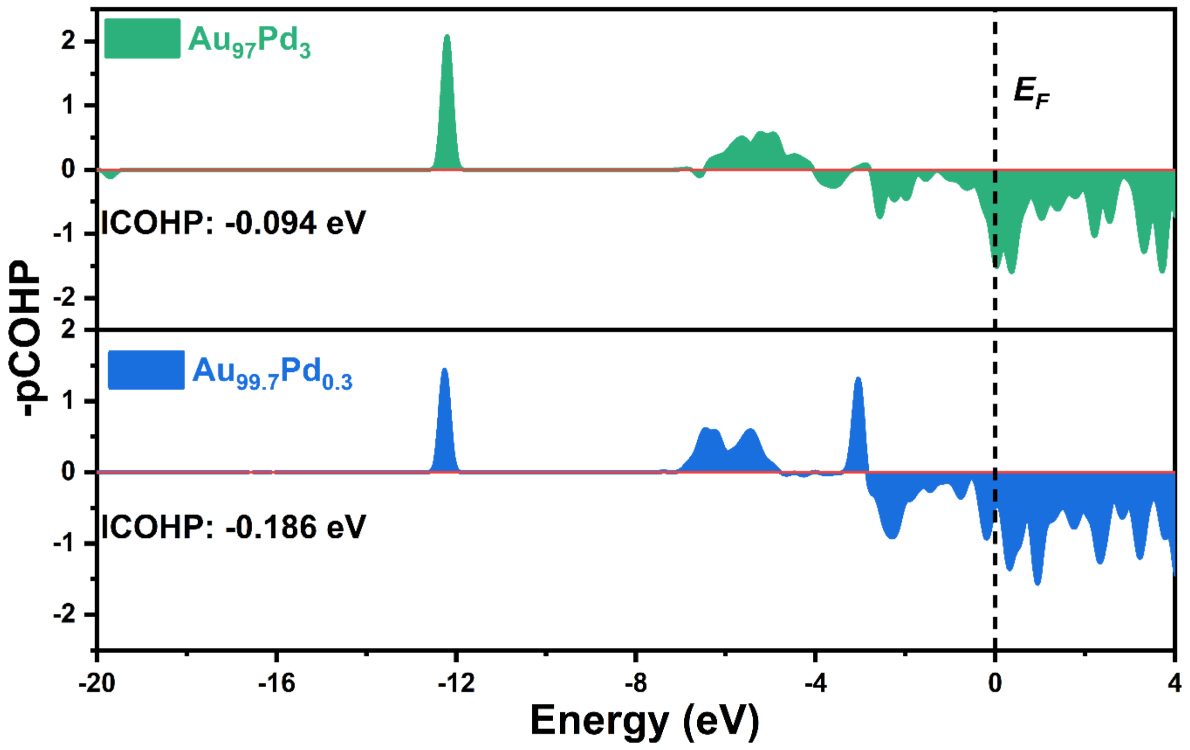

Figure S25. COHP bonding analysis of Pd-N interactions (Pd at the surface site and N in the adsorbed NO)

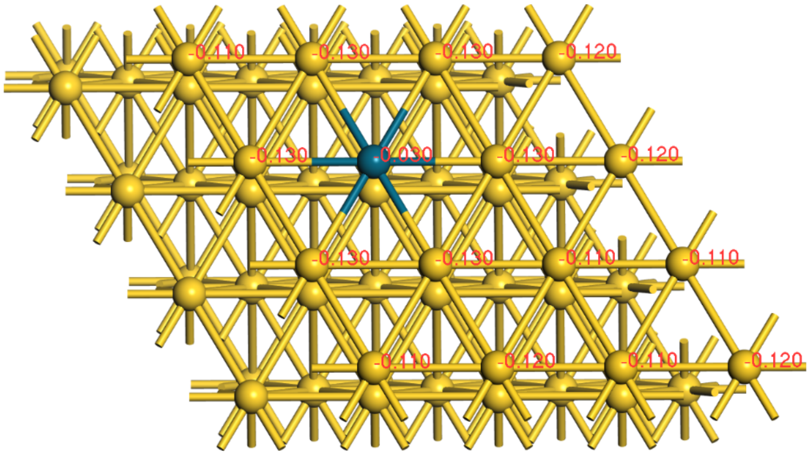

Figure S26. The Mulliken charge analysis of AuPd alloy in $Au_{99.7}Pd_{0.3}$ NPs model under an applied voltage (0.5 V $Å^{-1}$)

Table S3. Mulliken charge distributions of AuPd alloy in $Au_{99.7}Pd_{0.3}$ NPs model under an applied voltage (0.5 V Å^{-1}), as shown in **Figure S10**.

**AuPt alloy shell in $Au_{99.7}Pd_{0.3}$ under voltage**				
Mulliken Charge	Pd	Au1	Au2	Au3
	-0.030	-0.130	-0.130	-0.130
	Au4	Au5	Au6	
	-0.130	-0.130	-0.130	

References

(1) Ciesielski, A.; Skowronski, L.; Trzcinski, M.; Szoplik, T. Controlling the Optical Parameters of Self-Assembled Silver Films with Wetting Layers and Annealing. *Appl Surf Sci* **2017**, *421*, 349–356. https://doi.org/10.1016/J.APSUSC.2017.01.039.

(2) Mitra, S.; Chattopadhyay, R.; Ghosh, J.; Bysakh, S.; Bhadra, S. K. Modelling of Measured Optical Properties of Pd–Au Alloy Ultrathin Film for Room Temperature Hydrogen Sensing. *physica status solidi (a)* **2016**, *213* (9), 2406–2413. https://doi.org/10.1002/PSSA.201600051.

(3) Feser, J.; Sobh, A. N. DDSCAT Convert: A Target Generation Tool. **2013**. https://doi.org/10.21981/W4GM-SD35.

(4) Draine, B. T.; Flatau, P. J. Discrete-Dipole Approximation For Scattering Calculations. *J. Opt. Soc. Am. A* **1994**, *11*, 1491.
